# On the best constants of Schur multipliers of second order divided difference functions

**DOI:** 10.1007/s00208-025-03111-y

**Published:** 2025-03-03

**Authors:** Martijn Caspers, Jesse Reimann

**Affiliations:** TU Delft, EWI/DIAM, P.O.Box 5031, 2600 GA Delft, The Netherlands

## Abstract

We give a new proof of the boundedness of bilinear Schur multipliers of second order divided difference functions, as obtained earlier by Potapov, Skripka and Sukochev in their proof of Koplienko’s conjecture on the existence of higher order spectral shift functions. Our proof is based on recent methods involving bilinear transference and the Hörmander–Mikhlin–Schur multiplier theorem. Our approach provides a significant sharpening of the known asymptotic bounds of bilinear Schur multipliers of second order divided difference functions. Furthermore, we give a new lower bound of these bilinear Schur multipliers, giving again a fundamental improvement on the best known bounds obtained by Coine, Le Merdy, Potapov, Sukochev and Tomskova. More precisely, we prove that for $f \in C^2({\mathbb {R}})$ and $1< p, p_1, p_2 < \infty$ with $\frac{1}{p} = \frac{1}{p_1} + \frac{1}{p_2}$ we have

$$\begin{aligned} \Vert M_{f^{[2]}}: S_{p_1} \times S_{p_2} \rightarrow S_p \Vert \lesssim \Vert f'' \Vert _\infty D(p, p_1, p_2), \end{aligned}$$

where the constant $D(p, p_1, p_2)$ is specified in Theorem 7.1 and $D(p, 2p, 2p) \approx p^4 p^*$ with $p^*$ the Hölder conjugate of *p*. We further show that for $f(\lambda ) = \lambda \vert \lambda \vert ,$ $\lambda \in {\mathbb {R}},$ for every $1< p < \infty$ we have

$$\begin{aligned} p^2 p^*\lesssim \Vert M_{f^{[2]}}: S_{2p} \times S_{2p} \rightarrow S_p \Vert . \end{aligned}$$

Here $f^{[2]}$ is the second order divided difference function of *f* with $M_{f^{[2]}}$ the associated Schur multiplier. In particular it follows that our estimate *D*(*p*, 2*p*, 2*p*) is optimal for $p \searrow 1.$

## Introduction

In [35], Potapov, Skripka and Sukochev resolved a fundamental open conjecture by Koplienko [26]. This conjecture asserts the existence of so-called *spectral shift functions* $\eta _{n,H,V},$ for which the expression

1.1 $$\begin{aligned} \textrm{Tr}\left( f(H+V)-\sum _{k=0}^{n-1}\frac{1}{k!} \frac{d^k}{dt^k}f(H+tV)\Bigr |_{t=0}\right) = \int _{{\mathbb {R}}}f^{(n)}(t)\eta _{n,H,V}(t)dt \end{aligned}$$

is well-defined for the trace $\textrm{Tr}$ on bounded operators on a Hilbert space, *H* a self-adjoint operator, and *V* in the Schatten class $S_n.$ The existence of the spectral shift function goes back to the fundamental work of Krein [27, 28] and Lifschitz [31], and has ample applications in perturbation theory, mathematical physics, and noncommutative geometry. See [18] for an overview.

The key result in [35] is [35, Remark 5.4], which is a direct consequence of the more general result proved in [35, Theorem 5.3]. It asserts that multiple operator integrals of higher order divided difference functions are bounded maps on Schatten classes. The precise statement of [35, Remark 5.4] in the second order case, up to the boundedness constant, is recorded below as Theorem A.

In the linear case, i.e. order one, the search for optimal proofs and constants for operator integrals of divided difference functions has attracted great attention and a considerable number of the most important problems have been solved. The existence of first order spectral shift functions was first resolved in [36], and soon after the proofs were optimised to yield sharp estimates for double operator integrals of divided difference functions. In particular, the best constants were found in [2], and weak-$L^1$ and ${\textrm{BMO}}$ end-point estimates have been obtained in [5, 6] respectively. Furthermore, in the range $0< p < 1,$ the boundedness of double operator integrals of divided difference functions has fully been clarified recently by McDonald and Sukochev [32]. For $p=1,$ the best known result goes back to Peller [34]. Finally, a rather general Hörmander–Mikhlin–Schur multiplier theorem was established in the groundbreaking work [12], yielding the main results of [2, 36] as a special case.

When we consider the higher order problem of finding good bounds on multilinear operator integrals of divided difference functions as in [35], nothing is known about optimal bounds or end-point estimates except for the case of the generalised absolute value map [7]. Since the key results from [35] were proven, which is over a decade ago, significant advances have been made in the theory of Schur multipliers. This motivates our re-examination of this result, as we investigate here whether recent proof methods offer new insights. Let us first state our main result and then comment on the proof methods.

**Upper bounds.** We first define the second order divided difference functions. Let $f \in C^2({\mathbb {R}}),$ then the first order divided difference function is defined by the difference quotient

$$\begin{aligned} f^{[1]}(\lambda ,\mu ):=\frac{f(\lambda )-f(\mu )}{\lambda -\mu } \end{aligned}$$

for $\lambda \ne \mu ,$ and by setting $f^{[1]}(\lambda ,\lambda ):= f'(\lambda ).$ The second order divided difference function is then defined by

$$\begin{aligned} f^{[2]}(\lambda _0,\lambda _1,\lambda _2):=\frac{f^{[1]} (\lambda _{i-1},\lambda _{i})-f^{[1]}(\lambda _{i},\lambda _{i+1})}{\lambda _{i-1}-\lambda _{i+1}} \end{aligned}$$

where *i* is chosen such that $\lambda _{i-1} \not = \lambda _{i+1},$ with $\lambda _3$ interpreted as $\lambda _0,$ and otherwise we set $f^{[2]}(\lambda ,\lambda ,\lambda ):= f''(\lambda ).$ The function $f^{[2]}$ is well-defined and invariant under permutation of the variables. Our main result is now stated as follows. The definition of a Schur multiplier will be recalled in Sect. 2. Throughout the paper we use the notation $p^*= \frac{p}{p-1}$ for the Hölder conjugate of $1< p < \infty .$

### Theorem A

For every $f \in C^2({\mathbb {R}})$ and for every $1<p, p_1, p_2 < \infty$ with $\frac{1}{p} = \frac{1}{p_1} + \frac{1}{p_2}$ we have

$$\begin{aligned} \Vert M_{f^{[2]}}: S_{p_1} \times S_{p_2} \rightarrow S_{p} \Vert \lesssim D(p, p_1, p_2) \Vert f'' \Vert _\infty , \end{aligned}$$

where

$$\begin{aligned}  &   D(p, p_1, p_2) = C(p,p_1, p_2) (\beta _{p_1} + \beta _{p_2} ) + \beta _{p_1} \beta _{p_2}(\beta _p + \beta _{p_1} + \beta _{p_2} ), \\  &   C(p,p_1, p_2) = \beta _{p}\beta _{p_1}\beta _{p_2} +\min (\beta _{p_1}^2\beta _{p},\beta _{p}^2\beta _{p_1}) + \min (\beta _{p_2}^2\beta _{p},\beta _{p}^2\beta _{p_2})\\  &   +\min (\beta _{p_2}^2\beta _{p_1},\beta _{p_1}^2\beta _{p_2}), \end{aligned}$$

and $$\beta _q = q q^*.$$

In particular, if we set $p_1=p_2=2p$ we get the following asymptotic behaviour for the constant *D*(*p*, 2*p*, 2*p*). For $p\rightarrow \infty ,$ *D*(*p*, 2*p*, 2*p*) is of order at most $O(p^4),$ and of order $O(p^*)$ when $p \searrow 1.$ To see the latter limit, just note that $(2p)^*\nearrow 2$ and in particular does not blow up. This improves on the constant obtained by the proof method in [35] by eight orders, see Remark 7.3. Note that in Theorem B below, we justify that our constants must be quite close to the optimal ones.

**Proof methods.** We now describe the novel parts of our proof. Essentially, there are four aspects: avoidance of triangular truncations, bilinear transference, the use of the Hörmander–Mikhlin–Schur multiplier theorem [12], and finally, in combining the estimates we use a range of bilinear multipliers that map to $S_1.$

To start with, our proof relies on the following decomposition of the divided difference function into two-variable terms and three-variable Toeplitz form terms.

1.2 $$\begin{aligned} f^{[2]}(\lambda _0, \lambda _1, \lambda _2) = \underbrace{ \frac{\lambda _0 - \lambda _1}{ \lambda _0 - \lambda _2} }_{\begin{array}{c} \text {Three-variable} \\ \text {Toeplitz term} \end{array}} \underbrace{ f^{[2]}(\lambda _0, \lambda _1,\lambda _1) }_{ \text {Two-variable term } } + \underbrace{ \frac{\lambda _1 - \lambda _2}{ \lambda _0 - \lambda _2} }_{\begin{array}{c} \text {Three-variable} \\ \text {Toeplitz term} \end{array}} \underbrace{ f^{[2]}(\lambda _1, \lambda _{1}, \lambda _{2} )}_{\text { Two-variable term } }.\nonumber \\ \end{aligned}$$

This yields a decomposition of the corresponding Schur multiplier into linear Schur multipliers and bilinear Toeplitz form Schur multipliers, which we can treat separately. Crucially, we refine the decomposition (1.2) such that we can avoid the use of triangular truncations. This alone improves the upper bound on the norm of the Schur multiplier by three orders in *p* compared to [35].

The boundedness of a linear Toeplitz form Schur multiplier is implied by the boundedness of an associated Fourier multiplier through the transference method [1, 3, 33]. This transference method was recently extended to multilinear Toeplitz form Schur multipliers [8, 9]. We apply this to reduce our proof of the boundedness of the bilinear Toeplitz form Schur multipliers to the boundedness of the associated bilinear Fourier multiplier.

For this, we use that it is possible to show that this Fourier multiplier is a so-called Calderón–Zygmund operator. Such operators are known to be well-behaved under extension to UMD spaces in the linear case, such as for example the Schatten classes $S_p,$ $p\in (1,\infty ),$ see e.g. the monograph [23]. This result was recently extended to multilinear Calderón–Zygmund operators [15]. Unfortunately, the proofs in [15] do not keep track of the constants, though following the proof gives an explicit constant. We have therefore carefully outlined the proof of [15] in the appendix of our paper, as the *p*-dependence of the bound when considering Schatten classes concerns our main result. A very important observation is that we are dealing in this paper with Calderón–Zygmund operators that are Fourier multipliers, hence the paraproducts appearing in the multilinear dyadic representation theorem used in [15] vanish. This also yields an improvement on the bounds of our Calderón–Zygmund operators.

For non-Toeplitz form Schur multipliers, the transference method is generally difficult to apply, if at all possible. However, a recent result on the boundedness of linear Schur multipliers, including those of non-Toeplitz form, gives a rather simple sufficient condition for their boundedness. In [12], it was shown that a Hörmander–Mihlin type condition implies boundedness of the Schur multipliers $M_m,$ even if the symbol *m* is not of Toeplitz form. In fact, a slightly weaker condition is sufficient, as mixed derivatives need not be considered. It turns out that these Hörmander–Mihlin type conditions can be used to effectively estimate the linear (non-Toeplitz) terms occurring in (1.2).

Finally we need to combine the estimates we get for the three-variable Toeplitz terms with the ones for the two variable terms. Each of these terms yield a constant of order $O(p^*)$ for $p \searrow 1$ and so a naive combination of the estimates would yield order $O( (p^*)^2).$ Interestingly, we have found a way to combine the two estimates so that for the asymptotics for $p \searrow 1$ only one of the terms is relevant, and we are able to control the norm of our Schur multiplier with order $O(p^*)$ again. For this we prove that certain bilinear multipliers that appear in our decomposition actually map boundedly to $S_1.$

**Lower bounds.** In the final part of this paper we establish a lower bound for the bilinear Schur multiplier appearing in Theorem A. An alternative form of this problem was already considered in [11], where it was shown that there exists a function $f \in C^2({\mathbb {R}})$ for which $M_{f^{[2]}}$ does not map $S_2 \times S_2$ to $S_1$ boundedly. Outside of $[-1, 1],$ this function is given by $f(s) = s \vert s\vert ,$ and it is $C^2$ inside $[-1, 1].$ Such functions are generalised versions of the absolute value map and have played an important role in perturbation theory ever since the results of Kato [24] and Davies [13] on Lipschitz properties of the absolute value map. A weak type estimate for generalised absolute value maps was obtained in [7].

We use the generalised absolute value function to provide lower bounds of bilinear Schur multipliers in the following way. Note that since this function is not $C^2,$ we make sense of the second order derivative as a weak derivative.

### Theorem B

Let $f(s) = s \vert s\vert , s \in {\mathbb {R}}.$ For every $1< p < \infty ,$ we have

1.3 $$\begin{aligned} \Vert M_{f^{[2]}}: S_{2p} \times S_{2p} \rightarrow S_{p} \Vert \gtrsim p^2 p^*. \end{aligned}$$

Our proof method is as follows. For Schur multipliers whose symbol is continuous on an open subset $\Omega \subseteq {\mathbb {R}}^2,$ restricting the symbol to any discrete subset $X \times Y \subseteq \Omega$ yields a new Schur multiplier whose norm is not larger than the norm of the original Schur multiplier, see [29, Theorem 1.19] for this restriction theorem. Further, Davies (see [13, Lemma 10]) showed that one can approximate the triangular truncation map by restrictions of the divided difference function of the usual absolute value map to discrete sets. Here we show that also for the second order divided difference function of the generalised absolute value map, we can find restrictions to discrete sets that approximate the triangular truncation map. In turn, sharp lower bounds for the triangular truncation map are known and go back to Krein’s analysis of singular values of the Volterra operator [19]. By combining these ideas, we are able to find good lower bounds for our bilinear Schur multipliers of second order divided difference functions. Remarkably, we obtain a square power $p^2$ for the asymptotics $p \rightarrow \infty$ and a linear term $p^*$ for $p \searrow 1.$

Theorem B closely relates to the main result of [11]; in fact it implies a mild variation of the main theorem of [11]. In Remark 8.5 we conceptually compare our proof to [11] and argue that it gives a fundamentally better lower bound than what the method from [11] would give.

Note in particular that for $p \searrow 1,$ Theorems A and B yield that the asymptotics of the norm of (1.3) for general *f* are precisely of order $O(p^*).$ The asymptotics for $p \rightarrow \infty$ are narrowed down to an order between $O(p^2)$ and $O(p^4),$ and both the lower and upper bounds we find here are fundamentally better than what was previously known.

**Structure of the paper.** Section 2 contains preliminaries on Schur multipliers and Calderón–Zygmund operators. In Sect. 3, we present a decomposition of the Schur multiplier of second order divided difference functions into linear terms and bilinear Toeplitz form terms. Their boundedness is shown in Sect. 4 (linear terms) and Sects. 5 and  6 (bilinear terms). In Sect. 7, we prove Theorem A, as well as an additional extrapolation result. Theorem B is proven in Sect. 8. In Appendix A we have incorporated all arguments that are needed to obtain the explicit constants of Theorem 6.5; this essentially requires a careful analysis of the proofs in [15] and references given there. We decided to give full details here as this contributes directly to our main result.

## Preliminaries

We recall the following preliminaries, for which we refer to [39] for multilinear operator integrals, to [21] for harmonic analysis, and to [20] for (scalar-valued) multilinear Calderón–Zygmund theory.

### General notation

We let the natural numbers ${\mathbb {N}}$ be all integer numbers greater than or equal to 1. We shall write $A \lesssim B$ for saying that expression *A* is always smaller than *B* up to an absolute constant, and $A\approx B$ for $A\lesssim B \lesssim A.$ We write $f=O(g)$ if we have $|f(\lambda )|\lesssim g(\lambda )$ as $\lambda$ approaches some specified limit (usually $\lambda \rightarrow \infty$). For $f \in C^n({\mathbb {R}})$ we let $f^{(n)}$ denote the *n*-th order derivative. We call a function *smooth* if it is a $C^{\infty }$-function on its domain. For a continuous function $f:D\rightarrow {\mathbb {C}},$ $D\subseteq {\mathbb {R}}^d,$ we define its *support* to be the closure of the set $\{x\in D\mid f(x)\ne 0\}.$ We let $C_c({\mathbb {R}})$ denote the continuous functions on ${\mathbb {R}}$ with compact support. The Fourier transform of a Schwartz function *m* is defined as

2.1 $$\begin{aligned} ({\mathcal {F}}m)(x) = (2\pi )^{-\frac{d}{2}} \int _{{\mathbb {R}}^d} m(\xi )e^{-i \xi \cdot x} d\xi . \end{aligned}$$

We extend ${\mathcal {F}}$ in the usual way to the space of tempered distributions.

For $p \in (1, \infty )$ we set $p^*= p/(p-1),$ which is the Hölder conjugate of *p*. The set $\Delta \subseteq {\mathbb {R}}^{3d}$ is the set of diagonal elements $(\lambda , \lambda , \lambda ),$ $\lambda \in {\mathbb {R}}^d;$ we shall often require this only for $d=1.$ We use notations like $\{ \lambda = \mu \}$ to denote the set $\{ (\lambda , \mu ) \in {\mathbb {R}}^2 \mid \lambda = \mu \}.$ The Euclidean norm of a vector $\xi \in {\mathbb {R}}^d$ is denoted by $\vert \xi \vert = (\sum _{i=}^d \vert \xi _i \vert ^2)^{\frac{1}{2}}.$

We call a function $\varphi : {\mathbb {R}}^d {\setminus } \{0 \} \rightarrow {\mathbb {C}}$ *homogeneous* if it is homogeneous of order 0,  i.e. if for every $r > 0,$ $\xi \in {\mathbb {R}}^d {\setminus } \{0 \}$ we have $\varphi (r \xi ) = \varphi (\xi ).$ Moreover, $\varphi$ is called *even* if $\varphi (-\xi ) = \varphi (\xi )$ and *odd* if $\varphi (-\xi ) = -\varphi (\xi ).$ We may define a function ${\mathbb {R}}^d {\setminus } \Delta \rightarrow {\mathbb {C}}$ to be homogeneous, even, and odd with precisely the same definitions.

### Function spaces

We let $C_b({\mathbb {R}})$ denote the complex valued continuous bounded functions on ${\mathbb {R}}.$ Furthermore, we let $L_{loc}^1({\mathbb {R}}^d)$ denote the locally integrable functions ${\mathbb {R}}^d \rightarrow {\mathbb {C}}.$ The Banach space of *p*-integrable functions ${\mathbb {R}}^d \rightarrow {\mathbb {C}}$ with norm ${\Vert f \Vert _p = (\int _{{\mathbb {R}}^d} \vert f(x)\vert ^p dx)^{\frac{1}{p}}}$ is denoted by $L^p({\mathbb {R}}^d).$

### Schatten classes

For $p \in [1, \infty ),$ $S_p({\mathbb {R}}^d)$ denotes the Schatten *p*-class of $B(L^2({\mathbb {R}}^d)),$ consisting of all compact operators $x \in B(L^2({\mathbb {R}}^d))$ for which $\Vert x \Vert _p = \textrm{Tr}(\vert x \vert ^p)^{1/p}$ is finite. Furthermore, $S_\infty ({\mathbb {R}}^d)$ denotes the compact operators in $B(L^2({\mathbb {R}}^d)).$ For $p = 2$ we may identify $S_2({\mathbb {R}}^d)$ linearly with $L^2({\mathbb {R}}^d \times {\mathbb {R}}^d).$ This way, a kernel $A \in L^2({\mathbb {R}}^d \times {\mathbb {R}}^d)$ corresponds to the operator $(A \xi )(t) = \int _{ {\mathbb {R}}^d } A(t,s) \xi (s) ds$ in $S_2({\mathbb {R}}^d).$ We shall mostly be concerned with $d=1$ and write $S_p = S_p({\mathbb {R}}).$ Note that for $1<p<\infty ,$ the dual space of $S_p$ is $S_{p^*},$ where $p^*$ is the Hölder conjugate of *p*.

### Schur multipliers

For $m \in L^\infty ({\mathbb {R}}^{2d}),$ the multiplication map $M_m: A \mapsto m A$ acts boundedly on $L^2({\mathbb {R}}^d \times {\mathbb {R}}^d)$ and hence on $S_2({\mathbb {R}}^d).$ Now let us consider $d=1$ and introduce multilinear Schur multipliers as follows. Let $m \in L^\infty ({\mathbb {R}}^{n+1}).$ Then by [10, Proposition 5] there exists a unique bounded linear map

$$\begin{aligned} M_m: S_2 \times \ldots \times S_2 \rightarrow S_2: (A_1, \ldots , A_n) \mapsto M_m(A_1, \ldots , A_n), \end{aligned}$$

where the kernel of $M_m(A_1, \ldots , A_n)$ is given by

$$\begin{aligned}  &   M_m(A_1, \ldots , A_n)(s_0,s_n) = \int _{{\mathbb {R}}^{n-1}} m(s_0, \ldots , s_n) A_1(s_0, s_1) \ldots A_n(s_{n-1}, s_n) ds_1 \\  &   \ldots ds_{n-1},\quad s_0, s_n \in {\mathbb {R}}. \end{aligned}$$

Moreover, this map is bounded by $\Vert m \Vert _\infty ;$ this follows from the Cauchy-Schwartz inequality as observed in [10, Proposition 5]. We recall that in the linear case the following elementary (in)equalities for $1< p < \infty$ hold

2.2 $$\begin{aligned} \Vert M_{m}: S_{p} \rightarrow S_{p} \Vert= &   \Vert M_{m}: S_{p^*} \rightarrow S_{p^*} \Vert , \nonumber \\ \Vert M_{m}: S_{2} \rightarrow S_{2} \Vert\le &   \Vert M_{m}: S_{p} \rightarrow S_{p} \Vert , \nonumber \\ \Vert M_{m}: S_{2} \rightarrow S_{2} \Vert= &   \Vert m \Vert _{L^\infty ({\mathbb {R}}^2)}. \end{aligned}$$

where the first equality follows from duality, the second from complex interpolation between *p* and $p^*,$ and the last from the fact that we identified $S_2$ with $L^2({\mathbb {R}} \times {\mathbb {R}})$ on which *m* acts as a multiplication operator.

We may similarly define Schur multipliers on discrete sets as follows. Let *X* be any set and let $\ell ^2(X)$ be the Hilbert space of square summable functions on *X*. For $p \in [1, \infty ),$ let $S_p(\ell ^2(X))$ be the Schatten $S_p$-space of $B(\ell ^2(X))$ consisting of all operators $x \in B(\ell ^2(X))$ for which the norm $\Vert x \Vert _p := \textrm{Tr}(\vert x \vert ^p)^{1/p}$ is finite. For $x \in X,$ let $p_x$ be the orthogonal projection onto the span of $\delta _x \in \ell ^2(X),$ where $\delta _x(x)=1$ and $\delta _x(y)=0$ for $y\ne x.$ Let $m \in \ell ^\infty (X \times X \times X)$ and consider the bilinear Schur multiplier

2.3 $$\begin{aligned} M_{m}: S_{2}(\ell ^2(X)) \times S_{2}(\ell ^2(X) )\rightarrow &   S_{2}(\ell ^2(X)) \nonumber \\ (x, y)\mapsto &   \sum _{\lambda _1, \lambda _2, \lambda _3 \in X} m(\lambda _1, \lambda _2, \lambda _3) p_{\lambda _1} x p_{\lambda _2} y p_{\lambda _3}. \end{aligned}$$

As before, this map is bounded by $\Vert m \Vert _\infty ,$ see [10, Proposition 5].

For sets *F*, *G* consider the disjoint union $X = F \cup G$ and let $p_F$ and $p_G$ be the orthogonal projections of $\ell ^2(X)$ onto $\ell ^2(F)$ and $\ell ^2(G)$ respectively. Define $S_p(\ell ^2(F), \ell ^2(G)) = p_F S_p(\ell ^2(X), \ell ^2(X)) p_G.$ Then by (2.3) we see that $M_m$ maps $S_2(\ell ^2(F), \ell ^2(G)) \times S_2(\ell ^2(G), \ell ^2(F))$ to $S_2(\ell ^2(F), \ell ^2(F)).$

In either the continuous or discrete case, let $1 \le p, p_1, \ldots , p_n < \infty$ with $p^{-1} = \sum _{i=1}^n p_i^{-1}.$ We may consider the restriction of $M_m$ where its *i*-th inputs are restricted to the space $S_2 \cap S_{p_i}.$ If this restriction takes values in $S_p$ and has a bounded multilinear extension to $S_{p_1} \times \ldots \times S_{p_n},$ then this extension, also denoted by $M_m,$ is called an $(p_1, \ldots , p_n)$-Schur multiplier. In the discrete case, our terminology is the same but with $S_r$ replaced by $S_r(\ell ^2(X)).$

### Divided difference functions

#### Definition 2.1

(*Divided difference functions*) Let $f\in C^n({\mathbb {R}}),$ $n\in {\mathbb {N}}_0.$ We define the *n-th order divided difference function* $f^{[n]}$ of *f* inductively as follows. The first order divided difference function is constructed as $f^{[0]}(\lambda _0) \;:=\; f(\lambda _0).$ Then we set

2.4 $$\begin{aligned}&f^{[n]}(\lambda _0,\ldots ,\lambda _n)\nonumber \\&\quad := {\left\{ \begin{array}{ll} \frac{f^{[n-1]}(\lambda _0,\dotsc ,\lambda _{j-1},\lambda _{j+1},\dotsc ,\lambda _{n})-f^{[n-1]}(\lambda _0,\dotsc ,\lambda _{i-1},\lambda _{i+1},\dotsc ,\lambda _n)}{\lambda _i-\lambda _j}, & \text {if }\lambda _i\ne \lambda _j \text { for some } i\ne j, \\ \frac{f^{(n)}(\lambda _0)}{n!}, &  \lambda _0=\dotsc =\lambda _n, \end{array}\right. } \end{aligned}$$

where $\lambda _0,\dotsc ,\lambda _n\in {\mathbb {R}}.$ For $\lambda ,\mu \in {\mathbb {R}},$ we set

$$\begin{aligned} f^{[n]}(\lambda ^{(k)},\mu ^{(n+1-k)}):=f^{[n]} (\underbrace{\lambda ,\dotsc ,\lambda }_{k\ \text {times}},\underbrace{\mu ,\dotsc ,\mu }_{\begin{array}{c} n+1-k\\ \text {times} \end{array}}). \end{aligned}$$

We shall use repeatedly that divided difference functions are invariant under permutation of the variables, which can be checked by induction from its definition (or see [14]).

#### Remark 2.2

For $n=2$ and $f(\lambda ) = \lambda \vert \lambda \vert$ we define $f^{[2]}$ in the same way as in Definition 2.1, except that we set $f^{[2]} (\lambda , \lambda , \lambda ) = 0.$ Note that this alternative definition is required, as *f* is not a $C^2$-function.

#### Remark 2.3

We have from e.g. [35, Lemma 5.1] that

2.5 $$\begin{aligned} \Vert f^{[n]} \Vert _\infty \le \frac{\Vert f^{(n)} \Vert _\infty }{n!}. \end{aligned}$$

### Fourier multipliers and Calderón–Zygmund operators

In analogy to the linear definition, we define a bilinear *Fourier multiplier* with symbol $m\in L^{\infty }({\mathbb {R}}^{d}\times {\mathbb {R}}^{d})$ as follows. For Schwartz functions $f_1, f_2$ on ${\mathbb {R}}^d,$ we set

$$\begin{aligned} T_m(f_1,f_2)(x):=\frac{1}{(2\pi )^{d}}\int _{{\mathbb {R}}^{d}\times {\mathbb {R}}^d}m(\xi _1,\xi _2)({\mathcal {F}}f_1)(\xi _1)({\mathcal {F}}f_2)(\xi _2)e^{i(\xi _1+\xi _2)\cdot x}d\xi . \end{aligned}$$

Note that as ${\mathcal {F}}f_1$ and ${\mathcal {F}}f_2$ are Schwartz, the integral is over an integrable function and hence this formula is well-defined.

We recall the following from [15], which we need only for $d=1.$ Let *T* be an bilinear operator defined by an integral kernel, i.e. there exists a function $K:{\mathbb {R}}^{3d}{\setminus }\Delta \rightarrow {\mathbb {C}}$ such that for compactly supported bounded measurable functions $f_1, f_2 \in L^\infty _c({\mathbb {R}}^d),$

$$\begin{aligned} \langle T(f_1,f_2),f_{3}\rangle = \int _{{\mathbb {R}}^{3d}}K(x_{3},x_1,x_{2})\prod _{j=1}^{3}f_j(x_j)dx \end{aligned}$$

whenever ${\textrm{supp}}f_i\cap {\textrm{supp}}f_j=\emptyset$ for some $i\ne j.$ Such an operator *T* is called a *Calderón–Zygmund operator* if there exists some $\alpha \in (0,1]$ and $C_{K}>0$ such that the following conditions hold:

- (Size condition) for all $x=(x_1,x_2,x_3)\in {\mathbb {R}}^{3d}{\setminus }\Delta ,$
  $$\begin{aligned} |K(x)|\le \frac{C_K}{(|x_1-x_2|+|x_1-x_3|)^{2d}}, \end{aligned}$$
- (Smoothness condition) for all $j=1,2,3,$
  $$\begin{aligned} |K(x)-K(x')|\le \frac{C_K|x_j-x_j'|^{\alpha }}{(|x_1-x_2|+|x_1-x_3|)^{2d+\alpha }} \end{aligned}$$
  holds whenever $x,x'\in {\mathbb {R}}^{3d}{\setminus }\Delta$ such that $x_i=x_i'$ for $i\ne j$ and
  $$\begin{aligned} 2|x_j-x_j'|\le \max (|x_1-x_2|,|x_1-x_3|), \end{aligned}$$
- (Boundedness) for some (equivalently, for all) exponents $p_1,p_2\in (1,\infty )$ and $q_{3}\in (1/2,\infty )$ such that $1/p_1+1/p_2=1/q_{3},$
  $$\begin{aligned} \Vert T(f_1,f_2)\Vert _{L^{q_{3}}({\mathbb {R}}^d)}\lesssim \Vert f_1\Vert _{L^{p_1}({\mathbb {R}}^d)}\Vert f_2\Vert _{L^{p_2}({\mathbb {R}}^d)}. \end{aligned}$$

## Decomposing second order divided difference functions

The aim of this section is to show that the bilinear Schur multiplier of the second order divided difference function $f^{[2]}$ admits a decomposition as sums of compositions of bilinear Schur multipliers that are independent of *f* and of Toeplitz form as well as linear Schur multipliers. Such decompositions appear already in [35], but we require a different decomposition that allows us to incorporate the application of triangular truncations into the bilinear part.

Let $\epsilon > 0$ be small and fixed. Define the sets

$$\begin{aligned} A_{1, \epsilon }= &   (-2\epsilon , \pi /2+2\epsilon ) \cup (\pi -2\epsilon , 3\pi /2+2\epsilon ),\\ A_{2, \epsilon }= &   (\pi /2+\epsilon , 3 \pi /4+\epsilon ) \cup ( 3\pi /2 + \epsilon , 7 \pi /4 + \epsilon ),\\ A_{3, \epsilon }= &   (3\pi /4 - \epsilon , \pi - \epsilon ) \cup (7 \pi /4 - \epsilon , 2 \pi - \epsilon ). \end{aligned}$$

For a point $\xi =(\xi _1, \xi _2) \in {\mathbb {R}}^2 {\setminus } \{ 0 \}$ and $A \subseteq {\mathbb {R}}$ we say $\text {arg} (\xi _1, \xi _2) \in A$ in case there exists $\theta \in A$ such that $\xi = (\cos (\theta ), \sin (\theta )).$ We cut ${\mathbb {R}}^2 {\setminus } \{ 0 \}$ into the following areas:

3.1 $$\begin{aligned} \Delta _{1, \epsilon }= &   \{ (\xi _1, \xi _2) \in {\mathbb {R}}^2 {\setminus } \{ 0 \} \mid \text {arg} (\xi _1, \xi _2) \in A_{1, \epsilon } \},\nonumber \\ \Delta _{2, \epsilon }= &   \{ (\xi _1, \xi _2) \in {\mathbb {R}}^2 {\setminus } \{ 0 \} \mid \text {arg} (\xi _1, \xi _2) \in A_{2, \epsilon } \},\nonumber \\ \Delta _{3, \epsilon }= &   \{ (\xi _1, \xi _2) \in {\mathbb {R}}^2 {\setminus } \{ 0 \} \mid \text {arg} (\xi _1, \xi _2) \in A_{3, \epsilon } \}. \end{aligned}$$

**Fig. 1:**
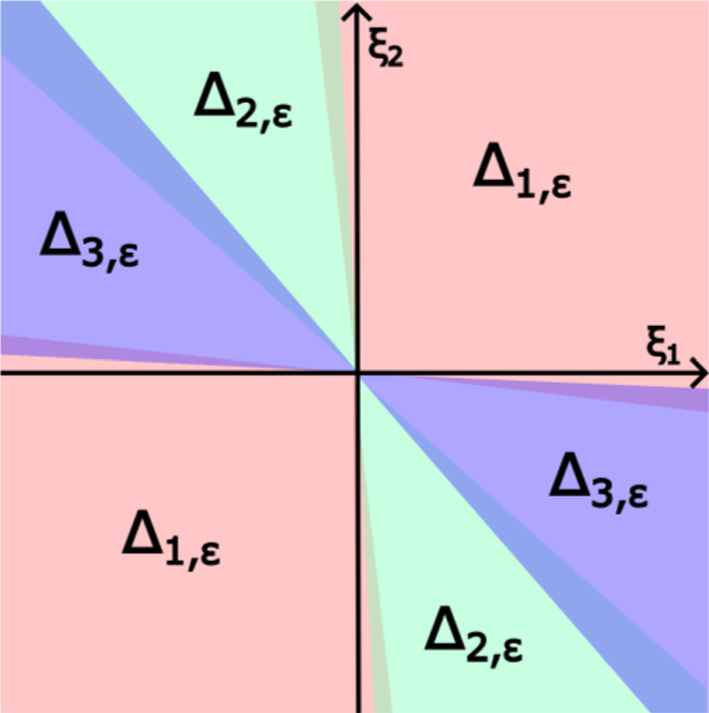
The sets $\Delta _{i,\varepsilon }$ as defined in (3.1). Note that the sets are partially overlapping

All these sets are radial in the sense that if $\xi \in \Delta _{j, \epsilon }$ then $r \xi \in \Delta _{j, \epsilon }$ for any $r >0.$ All $\Delta _{j, \epsilon }$ are open and satisfy $-\Delta _{j, \epsilon } = \Delta _{j, \epsilon }.$ Further, the sets $\Delta _{j, \epsilon },$ $j =1,2,3$ cover ${\mathbb {R}}^2 {\setminus } \{ 0 \}.$ See Fig. 1 for an illustration.

Let ${\mathbb {T}} \subseteq {\mathbb {R}}^2$ be the unit circle. Let $\theta _1', \theta _2', \theta _3': {\mathbb {T}} \rightarrow [0,1]$ be a partition of unity of the sets $\Delta _{j, \epsilon } \cap {\mathbb {T}},$ $j = 1,2,3.$ We may assume without loss of generality that the support of $\theta _j'$ is contained in $\Delta _{j, \epsilon } \cap {\mathbb {T}}.$ Furthermore, we may replace $\theta _j'(\xi ),$ $\xi \in {\mathbb {T}},$ by $\frac{1}{2}(\theta _j'(\xi ) + \theta _j'(-\xi ))$ and may therefore assume without loss of generality that $\theta _j'(\xi ) = \theta _j'(-\xi ).$ Set $\theta _j(\xi ) := \theta _j'(\xi /\vert \xi \vert )$ for $\xi \in {\mathbb {R}}^2 \backslash \{ 0 \}.$ Then $\theta _1, \theta _2, \theta _3: {\mathbb {R}}^2 {\setminus } \{ 0 \} \rightarrow [0,1]$ are smooth, even, homogeneous functions such that $\theta _1 + \theta _2 + \theta _3 = 1$ on ${\mathbb {R}}^2 {\setminus } \{ 0 \}$ and such that the support of $\theta _j$ is contained in $\Delta _{j, \epsilon }.$

Let

3.2 $$\begin{aligned} {\widetilde{\theta }}_j(\lambda _0, \lambda _1, \lambda _2) = \theta _j( \lambda _1 - \lambda _0, \lambda _2 - \lambda _1 ), \quad (\lambda _1, \lambda _2, \lambda _3) \in {\mathbb {R}}^3 {\setminus } \Delta , \end{aligned}$$

where we recall $\Delta = \{ (\lambda , \lambda , \lambda ) \mid \lambda \in {\mathbb {R}} \}.$ We obtain for $(\lambda _0,\lambda _1, \lambda _2) \in {\mathbb {R}}^3 {\setminus } \Delta$ and $f \in C^2({\mathbb {R}})$ that

3.3 $$\begin{aligned} f^{[2]}(\lambda _0, \lambda _1, \lambda _2) = \sum _{j=1}^3 {\widetilde{\theta }}_j(\lambda _0, \lambda _1, \lambda _2) f^{[2]}(\lambda _0, \lambda _1, \lambda _2). \end{aligned}$$

We shall now decompose each of these three summands. A general decomposition method can be found in [35, Lemma 5.8]; however, in the special case of divided difference functions, both the statement and the proof are more straightforward in our version below.

### Lemma 3.1

Let $f \in C^n({\mathbb {R}}),$ $n \ge 1$ and let $\lambda _0, \ldots , \lambda _n \in {\mathbb {R}}$ be such that for some $i,j \in \{ 0, \ldots , n \}$ we have $\lambda _i \not = \lambda _j.$ Let $\mu \in {\mathbb {R}}.$ Then, 

$$\begin{aligned} f^{[n]}(\lambda _0, \ldots , \lambda _n)= &   \frac{\lambda _i - \mu }{ \lambda _i - \lambda _j} f^{[n]}(\lambda _0, \ldots , \lambda _{j-1}, \mu , \lambda _{j+1}, \ldots , \lambda _n )\\  &   + \frac{\mu - \lambda _j}{ \lambda _i - \lambda _j} f^{[n]}(\lambda _0, \ldots , \lambda _{i-1}, \mu , \lambda _{i+1}, \ldots , \lambda _n ). \end{aligned}$$

### Proof

Since $f^{[n]}$ is invariant under permutation of its variables [14], we assume without loss of generality that $(i,j)=(0,1)$ to simplify the notation. It follows for $\mu \ne \lambda _i,$ $i=0,1,$ that

$$\begin{aligned}&f^{[n]}(\lambda _0,{\lambda }_1,\lambda _2,{\dotsc },\lambda _n) \\&\quad = \frac{1}{\lambda _0{-}\lambda _1}\left( f^{[n{-}1]}(\lambda _0, \lambda _2,\lambda _3,{\dotsc },\lambda _n){-}f^{[n{-}1]} ({\lambda }_1,{\lambda }_2,{\dotsc },{\lambda }_n)\right) \\&\quad =\frac{1}{\lambda _0-\lambda _1}\left( f^{[n-1]} (\lambda _0,\lambda _2,\lambda _3,\dotsc ,\lambda _n) -f^{[n-1]}(\mu ,\lambda _2,\lambda _3,\dotsc ,\lambda _n)\right) \\&\qquad + \frac{1}{\lambda _0-\lambda _1}\left( f^{[n-1]} (\mu ,\lambda _2,\lambda _3,\dotsc ,\lambda _n)-f^{[n-1]} (\lambda _1,\lambda _2,\dotsc ,\lambda _n)\right) \\&\quad =\frac{\lambda _0-\mu }{\lambda _0-\lambda _1} f^{[n]}(\lambda _0,\mu ,\lambda _2,\lambda _3,\dotsc ,\lambda _n) +\frac{\mu -\lambda _1}{\lambda _0-\lambda _1}f^{[n]}(\mu , \lambda _1,\lambda _2,\dotsc ,\lambda _n). \end{aligned}$$

Note the same formula holds for $\lambda _0 = \mu$ or $\lambda _1=\mu$ as long as $\lambda _0\ne \lambda _1.$ Indeed, assume without loss of generality $\lambda _0=\mu \ne \lambda _1,$ then

$$\begin{aligned}  &   \underbrace{\frac{\lambda _0-\mu }{\lambda _0-\lambda _1}}_{=0}f^{[n]}(\lambda _0,\mu ,\lambda _2,\lambda _3,\dotsc ,\lambda _n) +\underbrace{\frac{\mu -\lambda _1}{\lambda _0-\lambda _1}}_{=1}f^{[n]}(\mu ,\lambda _1,\lambda _2,\dotsc ,\lambda _n)\\  &   = f^{[n]}(\lambda _0,\lambda _1,\lambda _2,\dotsc ,\lambda _n). \end{aligned}$$

$$\square $$

We define the following functions for $(\lambda _0, \lambda _1, \lambda _2) \in {\mathbb {R}}^3.$ Let

$$\begin{aligned} \psi _1(\lambda _0, \lambda _1, \lambda _2)= &   \frac{\lambda _0 - \lambda _1}{\lambda _0 - \lambda _2}, \quad \lambda _0 \not = \lambda _2, \\ \psi _2(\lambda _0, \lambda _1, \lambda _2)= &   \psi _1(\lambda _2, \lambda _0, \lambda _1) = \frac{\lambda _2 - \lambda _0}{\lambda _2 - \lambda _1}, \quad \lambda _2 \not = \lambda _1, \\ \psi _3(\lambda _0, \lambda _1, \lambda _2)= &   \psi _1(\lambda _1, \lambda _2, \lambda _0) = \frac{\lambda _1 - \lambda _2}{\lambda _1 - \lambda _0}, \quad \lambda _0 \not = \lambda _1. \end{aligned}$$

and

$$\begin{aligned} \phi _f(\lambda , \mu ) = f^{[2]}(\lambda , \mu , \mu ), \quad \mathring{\phi }_f(\lambda , \mu ) = f^{[2]}(\lambda , \lambda , \mu ), \quad \lambda , \mu \in {\mathbb {R}}. \end{aligned}$$

As divided difference functions are permutation invariant, we have $\mathring{\phi }_f(\lambda , \mu ) = \phi _f(\mu , \lambda ).$

At this point we note that ${\widetilde{\theta }}_j \psi _j, j =1,2,3$ extends to a bounded continuous function on ${\mathbb {R}}^3 {\setminus } \Delta .$ Indeed, let $\lambda \in {\mathbb {R}}^3 {\setminus } \Delta$ be in the support of ${\widetilde{\theta }}_j.$ Note that the support is by definition a closed set contained in $\Delta _{j,\varepsilon },$ and that $\Delta _{j,\varepsilon }$ does not intersect the rays $\lambda _0 = \lambda _2$ (for $j=1$), $\lambda _2 = \lambda _1$ (for $j=2$), or $\lambda _0 = \lambda _1$ (for $j=3$), see (3.2). Hence ${\widetilde{\theta }}_j \psi _j$ is bounded on the support of ${\widetilde{\theta }}_j.$ We may thus extend ${\widetilde{\theta }}_j \psi _j$ by setting it equal to zero outside the support of ${\widetilde{\theta }}_j.$ This extended function is a smooth even homogeneous function on ${\mathbb {R}}^3 {\setminus } \Delta .$

We now apply the decomposition of Lemma 3.1 in the case $n=2.$ In case $(\lambda _0, \lambda _1, \lambda _2) \in \Delta _{1, \epsilon }$ we have, as also noted in the previous paragraph, that $\lambda _0 \not = \lambda _2,$ and we get

3.4 $$\begin{aligned} f^{[2]}(\lambda _0, \lambda _1, \lambda _2)= &   \frac{\lambda _0 - \lambda _1}{\lambda _0 - \lambda _2} f^{[2]}(\lambda _0, \lambda _1, \lambda _1) +\frac{\lambda _1 - \lambda _2}{\lambda _0 - \lambda _2} f^{[2]}(\lambda _1, \lambda _1, \lambda _2) \nonumber \\= &   \psi _1(\lambda _0, \lambda _1, \lambda _2) \phi _f(\lambda _0, \lambda _1) + (1-\psi _1)(\lambda _0, \lambda _1, \lambda _2) \mathring{\phi }_f(\lambda _1, \lambda _2).\nonumber \\ \end{aligned}$$

Similarly, we may use the permutation invariance of divided difference functions and find for $(\lambda _0, \lambda _1, \lambda _2) \in \Delta _{2, \epsilon }$ that

3.5 $$\begin{aligned}  &   f^{[2]}(\lambda _0, \lambda _1, \lambda _2)\nonumber \\  &   \quad = f^{[2]}(\lambda _2, \lambda _0, \lambda _1) = \frac{\lambda _2 - \lambda _0}{\lambda _2 - \lambda _1} f^{[2]}(\lambda _2, \lambda _0, \lambda _0) + \frac{\lambda _0 - \lambda _1}{\lambda _2 - \lambda _1} f^{[2]}(\lambda _0, \lambda _0, \lambda _1)\nonumber \\  &   \quad = \psi _2(\lambda _0, \lambda _1, \lambda _2) \mathring{\phi }_f(\lambda _0, \lambda _2) + (1-\psi _2)(\lambda _0, \lambda _1, \lambda _2) \mathring{\phi }_f(\lambda _0, \lambda _1). \end{aligned}$$

Finally, for $(\lambda _0, \lambda _1, \lambda _2) \in \Delta _{3, \epsilon },$ we have that

3.6 $$\begin{aligned}  &   f^{[2]}(\lambda _0, \lambda _1, \lambda _2)\nonumber \\  &   \quad = f^{[2]}(\lambda _1, \lambda _2, \lambda _0) = \frac{\lambda _1 - \lambda _2}{\lambda _1 - \lambda _0} f^{[2]}(\lambda _1, \lambda _2, \lambda _2) + \frac{\lambda _2 - \lambda _0}{\lambda _1 - \lambda _0} f^{[2]}(\lambda _2, \lambda _2, \lambda _0) \nonumber \\  &   \quad = \psi _3(\lambda _0, \lambda _1, \lambda _2) \phi _f(\lambda _1, \lambda _2) + (1-\psi _3)(\lambda _0, \lambda _1, \lambda _2) \phi _f(\lambda _0, \lambda _2). \end{aligned}$$

Combining (3.3), (3.4), (3.5), and (3.6) we find that

3.7 $$\begin{aligned}  &   f^{[2]}(\lambda _0, \lambda _1, \lambda _2)\nonumber \\  &   \quad = {\widetilde{\theta }}_1(\lambda _0, \lambda _1, \lambda _2) \left( \psi _1(\lambda _0, \lambda _1, \lambda _2) \phi _f(\lambda _0, \lambda _1) + (1-\psi _1)(\lambda _0, \lambda _1, \lambda _2) \mathring{\phi }_f(\lambda _1, \lambda _2) \right) \nonumber \\  &   \qquad + {\widetilde{\theta }}_2(\lambda _0, \lambda _1, \lambda _2) \left( \psi _2(\lambda _0, \lambda _1, \lambda _2) \mathring{\phi }_f(\lambda _0, \lambda _2) + (1-\psi _2)(\lambda _0, \lambda _1, \lambda _2) \mathring{\phi }_f(\lambda _0, \lambda _1) \right) \nonumber \\  &   \qquad + {\widetilde{\theta }}_3(\lambda _0, \lambda _1, \lambda _2) \left( \psi _3(\lambda _0, \lambda _1, \lambda _2) \phi _f(\lambda _1, \lambda _2) + (1-\psi _3)(\lambda _0, \lambda _1, \lambda _2) \phi _f(\lambda _0, \lambda _2) \right) .\nonumber \\ \end{aligned}$$

This decomposition (3.7) is not yet optimal for our purposes. In Sect. 6, we shall require that the symbols of the bilinear Toeplitz form Schur multipliers in our decomposition are odd (instead of even) homogeneous. This in particular implies the vanishing of the paraproduct terms that occur in transference methods for the bilinear term, improving the bound on the norm of our Schur multiplier. In order to achieve this, we include an extra sign function in the three-variable terms, for which we compensate by including a sign function in the two-variable terms. Set

$$\begin{aligned} \begin{array}{ll} \epsilon (\lambda , \mu )= \textrm{sign}(\mu - \lambda ),& \quad \epsilon _1(\lambda _0, \lambda _1, \lambda _2) = {{\,\textrm{sign}\,}}(\lambda _1 - \lambda _0),\\ \epsilon _2(\lambda _0, \lambda _1, \lambda _2) = {{\,\textrm{sign}\,}}(\lambda _2 - \lambda _1),& \quad \epsilon _3(\lambda _0, \lambda _1, \lambda _2) = {{\,\textrm{sign}\,}}(\lambda _2 - \lambda _0), \end{array} \end{aligned}$$

where we use the convention that ${{\,\textrm{sign}\,}}(0) = 1.$ Then we obtain the following decomposition, that we record here as a proposition.

### Proposition 3.2

Let $f \in C^2({\mathbb {R}})$ and let $(\lambda _0, \lambda _1, \lambda _2) \in {\mathbb {R}}^3 {\setminus } \Delta .$ Then, 

$$\begin{aligned} f^{[2]}(\lambda _0, \lambda _1, \lambda _2)= &   \epsilon _1(\lambda _0, \lambda _1, \lambda _2) {\widetilde{\theta }}_1(\lambda _0, \lambda _1, \lambda _2) \psi _1(\lambda _0, \lambda _1, \lambda _2) \, \cdot \, \epsilon (\lambda _0, \lambda _1) \phi _f(\lambda _0, \lambda _1) \\  &   + \epsilon _2(\lambda _0, \lambda _1, \lambda _2) {\widetilde{\theta }}_1(\lambda _0, \lambda _1, \lambda _2) (1-\psi _1)(\lambda _0, \lambda _1, \lambda _2) \, \cdot \, \epsilon (\lambda _1, \lambda _2) \mathring{\phi }_f(\lambda _1, \lambda _2) \\  &   + \epsilon _3(\lambda _0, \lambda _1, \lambda _2) {\widetilde{\theta }}_2(\lambda _0, \lambda _1, \lambda _2) \psi _2(\lambda _0, \lambda _1, \lambda _2) \, \cdot \, \epsilon (\lambda _0, \lambda _2) \mathring{\phi }_f(\lambda _0, \lambda _2) \\  &   + \epsilon _1(\lambda _0, \lambda _1, \lambda _2) {\widetilde{\theta }}_2(\lambda _0, \lambda _1, \lambda _2) (1-\psi _2)(\lambda _0, \lambda _1, \lambda _2) \, \cdot \, \epsilon (\lambda _0, \lambda _1) \mathring{\phi }_f(\lambda _0, \lambda _1) \\  &   + \epsilon _2(\lambda _0, \lambda _1, \lambda _2) {\widetilde{\theta }}_3(\lambda _0, \lambda _1, \lambda _2) \psi _3(\lambda _0, \lambda _1, \lambda _2) \, \cdot \, \epsilon (\lambda _1, \lambda _2) \phi _f(\lambda _1, \lambda _2)\\  &   + \epsilon _3(\lambda _0, \lambda _1, \lambda _2) {\widetilde{\theta }}_3(\lambda _0, \lambda _1, \lambda _2) (1-\psi _3)(\lambda _0, \lambda _1, \lambda _2) \, \cdot \, \epsilon (\lambda _0, \lambda _2) \phi _f(\lambda _0, \lambda _2). \end{aligned}$$

### Remark 3.3

In the previous expression we separated the two-variable terms from the three-variable terms with a ‘$\cdot$’.

For the corresponding Schur multipliers we find the following decomposition.

### Proposition 3.4

Let $f \in C^2({\mathbb {R}}).$ For $x,y \in S_2$ we have

3.8 $$\begin{aligned} M_{f^{[2]}}(x,y)= &   M_{ \epsilon _1 {\widetilde{\theta }}_1 \psi _1 }( M_{\epsilon \phi _f}(x), y ) + M_{ \epsilon _2 {\widetilde{\theta }}_1 (1-\psi _1) }( x, M_{\epsilon \mathring{\phi }_f }( y) ) \nonumber \\  &   + M_{\epsilon \mathring{\phi }_f}( M_{ \epsilon _3 {\widetilde{\theta }}_2 \psi _2 }( x, y ) ) + M_{ \epsilon _1 {\widetilde{\theta }}_2 (1-\psi _2) }( M_{ \epsilon \mathring{\phi }_f }( x), y ) \nonumber \\  &   + M_{ \epsilon _{2} {\widetilde{\theta }}_3 \psi _3 }( x, M_{\epsilon \phi _f}( y ) ) + M_{\epsilon \phi _f}( M_{ \epsilon _3 {\widetilde{\theta }}_3 (1-\psi _3) }( x, y ) ). \end{aligned}$$

### Proof

Note by Sect. 2.4 (or [10, Proposition 5]) that all linear and bilinear Schur multipliers appearing in (3.8) are bounded as maps on $S_2 \rightarrow S_2$ or $S_2 \times S_2 \rightarrow S_2.$ The proposition is now a consequence of a mild variation of [35, Lemma 3.2], which can easily be verified directly in the same way. $\square$

Now we outline our proof strategy for the next sections. All the linear Schur multipliers appearing in the decomposition (3.8) shall be estimated in Sect. 4. Each of the six summands in (3.8) contains a bilinear Schur multiplier. The last four of these summands shall be estimated in Sect. 5. The first two summands shall be estimated in Sect. 6. In fact, the methods of Sect. 6 can be used to estimate all six bilinear terms in (3.8). However, the constants obtained in Sect. 5 have better asymptotics, which is particularly relevant for the asymptotics for $p \searrow 1$ (as in Theorem A) for the third and sixth summand.

Strictly speaking, the sign functions $\epsilon ,$ $\epsilon _i$ in the last four summands of (3.8) are not needed for the estimates in Sect. 5. We have included them to show that these terms can also be estimated with the methods of Sect. 6.

## Bounding linear terms with the Hörmander–Mikhlin–Schur multiplier theorem

In this section, we show the boundedness of the linear Schur multipliers $M_{\phi _f}$ and $M_{\mathring{\phi }_f}$ defined in Sect. 3. Note that while the majority of this paper is concerned with second order divided difference functions, we will prove the results in this section for general *n*-th order divided difference functions.

We will use the following theorem.

### Theorem 4.1

[12, Theorem A] Let $\phi \in C^{\lfloor \tfrac{d}{2}\rfloor +1}({\mathbb {R}}^{2d}{\setminus }\{\lambda =\mu \}),$ $p\in (1,\infty ),$ and let $M_\phi$ be the Schur multiplier associated with $\phi .$ Then

$$\begin{aligned} \Vert M_\phi \Vert _{S_p\rightarrow S_p} \lesssim p p^*|\!|\!|\phi |\!|\!|_{\textrm{HMS}} \end{aligned}$$

with $$|\!|\!|\phi |\!|\!|_{\textrm{HMS}}:=\sum _{|\gamma |\le \lfloor \tfrac{d}{2}\rfloor +1}\Vert (\lambda ,\mu )\mapsto |\lambda -\mu |^{|\gamma |}(|\partial _\lambda ^{\gamma }\phi (\lambda ,\mu )|+|\partial _\mu ^{\gamma }\phi (\lambda ,\mu )|)\Vert _{\infty }.$$

We want to apply Theorem 4.1 to multipliers with symbol $\phi _f(\lambda ,\mu )=f^{[n]}(\lambda ^{(k)},\mu ^{(n+1-k)})$ for some $1\le k\le n.$ Here, we use the notation introduced in Sect. 2.5. We need the following two lemmas.

### Lemma 4.2

Let $n\ge 1,$ $0\le k \le n+1,$ and let $f\in C^{n+1}({\mathbb {R}}).$ Then the partial derivatives of the map $(\lambda ,\mu )\mapsto f^{[n]}(\lambda ^{(k)},\mu ^{(n+1-k)})$ are given by

$$\begin{aligned}&\partial _\lambda f^{[n]}(\lambda ^{(k)},\mu ^{(n+1-k)})=kf^{[n+1]}(\lambda ^{(k+1)},\mu ^{(n+1-k)}), \\&\partial _\mu f^{[n]}(\lambda ^{(k)},\mu ^{(n+1-k)})=(n+1-k)f^{[n+1]}(\lambda ^{(k)},\mu ^{(n+2-k)}). \end{aligned}$$

Furthermore,  $$\left( (\lambda ,\mu )\mapsto f^{[n]}(\lambda ^{(k)},\mu ^{(n+1-k)})\right) \in C^{1}({\mathbb {R}}^2{\setminus }\{ \lambda =\mu \}).$$

### Proof

Since $f^{[n]}$ is invariant under permutation of its variables, it is sufficient to calculate the partial derivatives in $\lambda .$ For $n=1,$ there are three cases to consider:

- $$k=0$$: $$\partial _\lambda f^{[1]}(\mu ,\mu )=0.$$
- $k=2$: $\partial _\lambda f^{[1]}(\lambda ,\lambda )=\partial _\lambda f'(\lambda )=f''(\lambda )=2f^{[2]}(\lambda ,\lambda ,\lambda ),$ where we used Definition 2.1.
- $k=1$: We use the product rule to show
  $$\begin{aligned} \partial _\lambda f^{[1]}(\lambda ,\mu )&=\partial _\lambda \frac{f(\lambda )-f(\mu )}{\lambda -\mu } = \frac{f'(\lambda )}{\lambda -\mu }-\frac{f(\lambda )-f(\mu )}{(\lambda -\mu )^2} \\&= \frac{f^{[1]}(\lambda ,\lambda )-f^{[1]}(\lambda ,\mu )}{\lambda -\mu }= f^{[2]}(\lambda ,\lambda ,\mu ). \end{aligned}$$

By definition, continuity of $f^{[1]}$ follows from continuity of *f*. Furthermore, its derivatives are continuous in $\lambda \ne \mu$ by continuity of $f''$ and $f^{[1]}.$

Now let $n\in {\mathbb {N}}.$ For $k=0,$ the statement is immediate. For $0<k\le n+1,$ we use the product rule and induction to show

$$\begin{aligned}&\partial _\lambda f^{[n]}(\lambda ^{(k)},\mu ^{(n+1-k)})\\&\quad =\frac{\partial _\lambda (f^{[n-1]}(\lambda ^{(k)},\mu ^{(n-k)}) - f^{[n-1]}(\lambda ^{(k-1)},\mu ^{(n+1-k)}))}{\lambda -\mu }\\&\qquad -\frac{f^{[n-1]}(\lambda ^{(k)},\mu ^{(n-k)}) - f^{[n-1]}(\lambda ^{(k-1)},\mu ^{(n+1-k)})}{(\lambda -\mu )^2} \\&\quad = \frac{k f^{[n]}(\lambda ^{(k+1)},\mu ^{(n-k)}) - (k-1) f^{[n]}(\lambda ^{(k)},\mu ^{(n+1-k)})-f^{[n]}(\lambda ^{(k)},\mu ^{(n+1-k)})}{\lambda -\mu } \\&\quad = kf^{[n+1]}(\lambda ^{(k+1)},\mu ^{(n+1-k)}). \end{aligned}$$

Continuity of $(\lambda ,\mu )\mapsto f^{[n]}(\lambda ^{(k)},\mu ^{(n+1-k)})$ in $\lambda \ne \mu$ follows by induction from continuity of the corresponding $f^{[n-1]}$-terms. As in the base case, continuity of its first derivatives in $\lambda \ne \mu$ follows from continuity of $f^{(n+1)}$ and $f^{[n]}.$ $\square$

### Lemma 4.3

For $$n\in {\mathbb {N}},$$ $$0\le k \le n+1,$$ $$0\le \gamma \le \min \{k,n+1-k\},$$ and $$(\lambda ,\mu )\in {\mathbb {R}}^2{\setminus }\{\lambda =\mu \},$$

$$\begin{aligned} |\lambda -\mu |^{\gamma }|\partial _\lambda ^{\gamma }f^{[n]}(\lambda ^{(k)},\mu ^{(n+1-k)})|\le 2^{\gamma }\frac{(k+\gamma -1)!}{(k-1)!}\frac{\Vert f^{(n)}\Vert _{\infty }}{n!}. \end{aligned}$$

### Proof

For $\gamma =0,$ this statement is immediate from (2.5). Let now $0<\gamma \le \min \{k,n+1-k\}.$ By repeatedly applying Lemma 4.2, we obtain

$$\begin{aligned} \partial _\lambda ^{\gamma }f^{[n]}(\lambda ^{(k)},\mu ^{(n+1-k)})=\frac{(k+\gamma -1)!}{(k-1)!}f^{[n+\gamma ]}(\lambda ^{(k+\gamma )},\mu ^{(n+1-k)}). \end{aligned}$$

We now decompose $f^{[n+\gamma ]}$ by applying the definition of divided difference functions multiple times as

$$\begin{aligned}&f^{[n+\gamma ]}(\lambda ^{(k+\gamma )},\mu ^{(n+1-k)})\\&\quad =\frac{1}{\lambda -\mu }\left( f^{[n+\gamma -1]}(\lambda ^{(k+\gamma )},\mu ^{(n-k)})- f^{[n+\gamma -1]}(\lambda ^{(k+\gamma -1)},\mu ^{(n+1-k)})\right) \\&\quad = \dotsc \\&\quad = \frac{1}{(\lambda -\mu )^\gamma } \sum _{j=0}^{\gamma } (-1)^j \left( {\begin{array}{c}\gamma \\ j\end{array}}\right) f^{[n]}(\lambda ^{(k+\gamma -j)},\mu ^{(n+1-k-(\gamma - j))}). \end{aligned}$$

Using the estimate $\Vert f^{[n]}\Vert _{\infty }\le \tfrac{\Vert f^{(n)}\Vert _{\infty }}{n!}$ from (2.5), we conclude

$$\begin{aligned}&|\lambda -\mu |^{\gamma }|\partial _\lambda ^{\gamma }f^{[n]}(\lambda ^{(k)},\mu ^{(n+1-k)})|\\&\quad \le \frac{(k+\gamma -1)!}{(k-1)!} \sum _{j=0}^{\gamma } \left( {\begin{array}{c}\gamma \\ j\end{array}}\right) |f^{[n]}(\lambda ^{(k+\gamma -j)},\mu ^{(n+1-k-(\gamma - j))})| \\&\quad \le \frac{(k+\gamma -1)!}{(k-1)!}\sum _{j=0}^{\gamma } \left( {\begin{array}{c}\gamma \\ j\end{array}}\right) \frac{\Vert f^{(n)}\Vert _{\infty }}{n!} =2^{\gamma } \frac{(k+\gamma -1)!}{(k-1)!}\frac{\Vert f^{(n)}\Vert _{\infty }}{n!}. \end{aligned}$$

$$\square $$

Altogether we can now show the following.

### Theorem 4.4

Let $n\in {\mathbb {N}},$ $f\in C^{n}({\mathbb {R}}),$ $1\le k\le n,$ and $p\in (1,\infty ).$ Set $\phi _f(\lambda ,\mu ):=f^{[n]}(\lambda ^{(k)},\mu ^{(n+1-k)}).$ Then

$$\begin{aligned} \Vert M_{\phi _f}\Vert _{S_p\rightarrow S_p}\lesssim \frac{2n+3}{n!} p p^*\Vert f^{(n)}\Vert _{\infty }. \end{aligned}$$

### Proof

We can apply Theorem 4.1, since $\phi _f\in C^{1}({\mathbb {R}}^2{\setminus }\{\lambda =\mu \})$ by Lemma 4.2. From Lemma 4.3, we conclude

$$\begin{aligned}&|\!|\!|\phi _f |\!|\!|_{\textrm{HMS}}\\&\quad \le \Vert \phi _f\Vert _{\infty }+\Vert (\lambda ,\mu )\mapsto |\lambda -\mu |\partial _\lambda \phi _f(\lambda ,\mu )\Vert _{\infty } +\Vert (\lambda ,\mu )\mapsto |\lambda -\mu |\partial _\mu \phi _f(\lambda ,\mu )\Vert _{\infty }\\&\quad \le (1 + 2k + 2(n+1-k))\frac{\Vert f^{(n)}\Vert _{\infty }}{n!}=\frac{2n+3}{n!}\Vert f^{(n)}\Vert _{\infty }. \end{aligned}$$

$$\square $$

### Remark 4.5

Recall that we set $\epsilon (\lambda , \mu ) = {{\,\textrm{sign}\,}}(\mu - \lambda ).$ Under the assumptions of Theorem 4.4 if follows also that

$$\begin{aligned} \Vert M_{\epsilon \phi _f}\Vert _{S_p\rightarrow S_p} \lesssim \frac{2n+3}{n!} p p^*\Vert f^{(n)}\Vert _{\infty }. \end{aligned}$$

Indeed, $\epsilon \phi _f$ satisfies the same Hörmander–Mikhlin differentiability criteria as $\phi _f ,$ so that we may appeal again to Theorem 4.1.

## Bilinear Schur multipliers that map to $S_1$

The aim of this section is to estimate the last four of the bilinear Schur multipliers occurring in the six summands of (3.4). It turns out that these Schur multipliers are special, as they admit an $S_1$-bound.

### Theorem 5.1

Let $m: {\mathbb {R}}^2 {\setminus } \{ 0 \} \rightarrow {\mathbb {C}}$ be smooth and homogeneous with support contained in one of the four quadrants $\sigma _1 {\mathbb {R}}_{>0} \times \sigma _2 {\mathbb {R}}_{>0} ,$ where $\sigma _j \in \{ +, -\}.$ Define ${\widetilde{m}}$ as in (3.2). Then for every $1 \le p < \infty ,$ $1< p_1, p_2 < \infty$ with $\frac{1}{p_1} +\frac{1}{p_2} = \frac{1}{p}$ we have

$$\begin{aligned} \Vert M_{{\widetilde{m}}}: S_{p_1} \times S_{p_2} \rightarrow S_p \Vert \lesssim C(m) p_1 p_1^*p_2 p_2^*, \end{aligned}$$

for a constant $C(m) > 0$ only depending on *m*.

### Proof

For simplicity assume that $\sigma _2 = +,$ as the other case can be treated similarly. Set then $\rho (\lambda ) = m(\lambda , 1),$ $\lambda \in {\mathbb {R}}.$ Then $\rho (\xi _1/\xi _2) = m(\xi _1/\xi _2, 1) = m(\xi _1, \xi _2),$ where the last equality follows as *m* is homogeneous and supported on $(\xi _1, \xi _2)$ with $\xi _2$ positive. Further, note once more that *m* is homogeneous and thus constant on rays. Since its support is a closed set contained in the quadrant $\sigma _1 {\mathbb {R}}_{>0} \times {\mathbb {R}}_{>0},$ it must thus be a proper radial subsector of that quadrant. Therefore, it follows that $\rho$ has compact support contained in $\sigma _1 (0, \infty ).$ In particular, $\rho$ is a compactly supported Schwartz function.

It follows that the function $t \mapsto \rho (\sigma _1 e^t),$ $t \in {\mathbb {R}}$ is Schwartz. So using Fourier inversion we write

$$\begin{aligned} \rho ( \sigma _1 e^t ) = \int _{\mathbb {R}} g(s) e^{ist} ds \end{aligned}$$

with *g* a Schwartz function. Substitute $t = \log (\sigma _1 \xi _1/\xi _2),$ where $(\xi _1, \xi _2) \in \sigma _1 {\mathbb {R}}_{>0} \times {\mathbb {R}}_{>0} .$ This gives

$$\begin{aligned} m(\xi _1, \xi _2) = \rho ( \frac{\xi _1}{\xi _2}) = \int _{\mathbb {R}} g(s) \vert \xi _1 \vert ^{is} \xi _2^{-is} ds, \quad (\xi _1, \xi _2) \in \sigma _1 {\mathbb {R}}_{>0} \times {\mathbb {R}}_{>0}. \end{aligned}$$

Let

$$\begin{aligned} k_s^1(\lambda _0, \lambda _1)= &   \left\{ \begin{array}{ll} \vert \lambda _1 - \lambda _0 \vert ^{is}, &  \quad \text {if } \sigma _1 (\lambda _1 - \lambda _0)>0, \\ 0, &  \quad \text {otherwise}. \end{array} \right. \\ k_s^2(\lambda _1, \lambda _2)= &   \left\{ \begin{array}{ll} (\lambda _2 - \lambda _1)^{is}, &  \quad \text {if } (\lambda _2 - \lambda _1) >0, \\ 0, &  \quad \text {otherwise}. \end{array} \right. \end{aligned}$$

Hence

$$\begin{aligned} {\widetilde{m}}(\lambda _0, \lambda _1, \lambda _2) = \int _{\mathbb {R}} g(s) k_s^1(\lambda _0, \lambda _1) k_{-s}^2(\lambda _1, \lambda _2) ds. \end{aligned}$$

It then follows that

$$\begin{aligned} M_{{\widetilde{m}} }(x,y) = \int _{\mathbb {R}} g(s) M_{k_s^1 }(x) M_{ k_{-s}^2 }(y) ds. \end{aligned}$$

Note that $|\!|\!|k_s^1 |\!|\!|_{\textrm{HMS}} = |\!|\!|k_s^2 |\!|\!|_{\textrm{HMS}} = 1+2 \vert s \vert .$ Thus by Theorem 4.1,

$$\begin{aligned} \Vert M_{{\widetilde{m}}}: S_{p_1} \times S_{p_2} \rightarrow S_p \Vert \lesssim&\int _{\mathbb {R}} g(s) (1+ 2\vert s \vert )^2 ds \, p_1 p_1^*p_2 p_2^*. \end{aligned}$$

This concludes the proof. $\square$

For the following corollary we recall the notation from Proposition 3.4.

### Corollary 5.2

Let $a_3 := \epsilon _3 {\widetilde{\theta }}_2 \psi _2,$ $a_4 := \epsilon _1 {\widetilde{\theta }}_2 (1 - \psi _2),$ $a_5 := \epsilon _2 {\widetilde{\theta }}_3 \psi _3$ and $a_6 := \epsilon _3 {\widetilde{\theta }}_3(1- \psi _3).$ Then for every $1 \le p< \infty , 1< p_1, p_2 < \infty$ with $\frac{1}{p_1} +\frac{1}{p_2} = \frac{1}{p}$ we have

$$\begin{aligned} \Vert M_{a_j}: S_{p_1} \times S_{p_2} \rightarrow S_p \Vert \lesssim C(a_j) p_1 p_1^*p_2 p_2^*, \quad 3 \le j \le 6, \end{aligned}$$

for a constant $C(a_j) >0$ only depending on $a_j.$

### Proof

Each of the functions $a_j$ is smooth, homogeneous, of Toeplitz form, and supported on one of the two quadrants $-\sigma {\mathbb {R}}_{>0} \times \sigma {\mathbb {R}}_{>0}$ with $\sigma \in \{+, -\}.$ Therefore the conclusion follows from Theorem 5.1. $\square$

### Remark 5.3

The constant $C(a_j)$ depends in particular on the choice of $\epsilon >0$ in Sect. 3, see (3.1). Note that we cannot expect a bound as in Corollary 5.2 that is uniform as $\epsilon \searrow 0,$ since in [8, Theorem 5.3] and its proof it is shown that such Schur multipliers do not map to $S_1.$

## Bilinear transference

The aim of this section is to estimate the remaining bilinear terms occurring in Proposition 3.4. The crucial observation is that these multipliers are of Toeplitz form and therefore, using bilinear transference techniques, can be estimated by Fourier multipliers and Calderón–Zygmund operators.

### Bilinear Calderón–Zygmund operators and Fourier multipliers

We say $K: {\mathbb {R}}^2 \rightarrow {\mathbb {C}}$ satisfies the *size condition* if for some constant $C_1>0$ we have

6.1 $$\begin{aligned} \vert K(z) \vert \le \frac{C_1}{\vert z \vert ^2}, \quad z \in {\mathbb {R}}^2 {\setminus } \{ 0 \}. \end{aligned}$$

We say that *K* satisfies the *smoothness condition* if *K* is continuously differentiable on ${\mathbb {R}}^2 {\setminus } \{ 0 \}$ and there exists some constant $C_2 > 0$ such that

6.2 $$\begin{aligned} \vert \nabla K(z) \vert \le \frac{C_2}{\vert z \vert ^3}, \quad z \in {\mathbb {R}}^2 {\setminus } \{ 0 \}. \end{aligned}$$

Set ${\widetilde{K}}(x,y,z) = K(x-y, x-z),$ $x,y,z \in {\mathbb {R}}.$ If *K* satisfies (6.1) and (6.2), then

6.3 $$\begin{aligned} \vert {\widetilde{K}}(x,y,z) \vert \le \frac{C_1}{ (\vert x-y \vert + \vert x - z \vert )^2}, \quad (x,y,z) \in {\mathbb {R}}^3 {\setminus } \Delta , \end{aligned}$$

and it follows from the chain rule that

6.4 $$\begin{aligned} \vert \nabla {\widetilde{K}}(x,y,z) \vert \le \frac{C_2}{ (\vert x-y \vert + \vert x - z \vert )^3}, \quad (x,y,z) \in {\mathbb {R}}^3 {\setminus } \Delta . \end{aligned}$$

It is assumingly well-known (see e.g. the introduction of [20]) that Condition 6.4 implies the following more general condition. We provide a proof for completeness as we did not find it in the literature.

#### Lemma 6.1

Suppose that *K* satisfies the smoothness condition. Let $(x_1,x_2,x_3) \in {\mathbb {R}}^3 {\setminus } \Delta .$ Let ${\widetilde{x}}_j \in {\mathbb {R}},$ $j =1,2,3,$ be such that

6.5 $$\begin{aligned} \vert x_j - {\widetilde{x}}_j \vert \le \frac{1}{2} \max ( \vert x_1 - x_2 \vert , \vert x_1 - x_3 \vert ). \end{aligned}$$

Then, 

$$\begin{aligned} \vert {\widetilde{K}}(x_1, x_2, x_3) - {\widetilde{K}}({\widetilde{x}}_1, x_2, x_3 ) \vert\lesssim &   \frac{ \vert x_1 - {\widetilde{x}}_1 \vert }{ (\vert x_1 - x_2 \vert + \vert x_1 - x_3 \vert )^3 },\\ \vert {\widetilde{K}}(x_1, x_2, x_3) - {\widetilde{K}}(x_1, {\widetilde{x}}_2, x_3 ) \vert\lesssim &   \frac{ \vert x_2 - {\widetilde{x}}_2 \vert }{ (\vert x_1 - x_2 \vert + \vert x_1 - x_3 \vert )^3 },\\ \vert {\widetilde{K}}(x_1, x_2, x_3) - {\widetilde{K}}(x_1, x_2, {\widetilde{x}}_3 ) \vert\lesssim &   \frac{ \vert x_3 - {\widetilde{x}}_3 \vert }{ (\vert x_1 - x_2 \vert + \vert x_1 - x_3 \vert )^3 }. \end{aligned}$$

#### Proof

We only prove the first estimate, the other two are proved in a similar way. It suffices to prove the case $x_2 \not = x_3,$ since ${\mathbb {R}}^3 {\setminus } \{ x_2 = x_3 \}$ is a dense subset of ${\mathbb {R}}^3 {\setminus } \Delta$ and *K* is continuous. Take $x_1'$ in the interval $[x_1, {\widetilde{x}}_1]$ (or in $[ {\widetilde{x}}_1, x_1]$ in case $x_1 > {\widetilde{x}}_1$ ) such that

$$\begin{aligned} \vert {\widetilde{K}}(x_1, x_2, x_3) - {\widetilde{K}}({\widetilde{x}}_1, x_2, x_3 ) \vert = \vert x_1 - {\widetilde{x}}_1 \vert \vert \partial _1 {\widetilde{K}} (x_1', x_2, x_3) \vert . \end{aligned}$$

But then the assumptions  (6.3) and (6.5) imply that

$$\begin{aligned} \vert {\widetilde{K}}(x_1, x_2, x_3) - {\widetilde{K}}({\widetilde{x}}_1, x_2, x_3 ) \vert&\lesssim \frac{ \vert x_1 - {\widetilde{x}}_1 \vert }{ (\vert {x_1'} - x_2 \vert + \vert x_1 - x_3 \vert )^3 } \\&\lesssim \frac{ \vert x_1 -{\widetilde{x}}_1 \vert }{ (\vert x_1 - x_2 \vert + \vert x_1 - x_3 \vert )^3 }, \end{aligned}$$

where the second inequality follows from (6.5) since

$$\begin{aligned}  &   |x_1-x_2| \le |x_1-x_1'|+|x_1'-x_2| \\    &   \quad \le |x_1-{\widetilde{x}}_1|+|x_1'-x_2| \\    &   \quad \le \frac{1}{2}|x_1-x_2| + |x_1'-x_2|. \end{aligned}$$

$$\square $$

Lemma 6.1 shows that the conditions (6.1) and (6.2) imply that the kernel ${\widetilde{K}}$ satisfies the size and smoothness conditions appearing in [15]. Next, we show that for bilinear Fourier multipliers with odd homogeneous symbols, their associated Calderón–Zygmund kernels satisfy these criteria. Recall that the Fourier transform ${\mathcal {F}}$ was defined in the preliminaries (2.1) in a distributional sense.

#### Proposition 6.2

Let $m: {\mathbb {R}}^2 {\setminus } \{ 0 \} \rightarrow {\mathbb {R}}$ be smooth and odd homogeneous,  and set ${m(0,0) = 0}.$ Then ${\mathcal {F}}m: {\mathbb {R}}^2 \rightarrow {\mathbb {C}}$ is a function satisfying conditions (6.1), (6.2), and ${({\mathcal {F}}m)(0,0)=0}.$

#### Proof

The proof is essentially the same as [5, Lemma 4.3] but for the convenience of the reader we give it here. We identify ${\mathbb {R}}^2$ with ${\mathbb {C}}.$ Since *m* is smooth on the circle, we may write

$$\begin{aligned} m(e^{i \theta }) = \sum _{k \in {\mathbb {Z}}} \alpha _k e^{ik \theta }, \quad \theta \in [0, 2\pi ), \end{aligned}$$

where the Fourier coefficients $\alpha _k$ decay faster than any polynomial. As *m* is odd, it has mean zero on the circle, and thus $\alpha _0 = 0.$ It follows that

$$\begin{aligned} m = \sum _{0 \not = k \in {\mathbb {Z}}} \alpha _k g_k, \quad g_k(z) = \frac{z^k}{\vert z \vert ^k}, \quad 0 \not = z \in {\mathbb {C}}. \end{aligned}$$

We have for $k \not = 0$ that $({\mathcal {F}} g_k)(0) = 0,$ and as in [5, Lemma 4.3] one can show that

$$\begin{aligned} ({\mathcal {F}} g_k)(z) = \frac{\vert k \vert }{2 \pi i^k} \frac{z^k}{\vert z \vert ^{k+2}}, \quad 0 \not = z \in {\mathbb {C}}. \end{aligned}$$

Hence $$({\mathcal {F}}m)(0) = 0$$ and

$$\begin{aligned} ({\mathcal {F}}m)(z) = \sum _{0 \not = k \in {\mathbb {Z}}} \frac{\vert k \vert \alpha _k }{ 2 \pi i^k} \frac{z^k}{\vert z \vert ^{k+2}}, \quad 0 \not = z \in {\mathbb {C}}. \end{aligned}$$

As the coefficients $\vert k \vert \alpha _k$ are summable it follows therefore that

$$\begin{aligned} \vert ({\mathcal {F}}m)(z) \vert \approx O(\vert z \vert ^{-2}), \quad \quad \vert \nabla ({\mathcal {F}}m)(z) \vert \approx O(\vert z \vert ^{-3}), \end{aligned}$$

which finishes the proof. $\square$

#### Proposition 6.3

Let $m: {\mathbb {R}}^2 {\setminus } \{ 0 \} \rightarrow {\mathbb {R}}$ be smooth,  odd,  homogeneous,  and set ${m(0,0) = 0}.$ Then the Fourier multiplier $T_m$ is a bilinear Calderón–Zygmund operator with kernel $-(2\pi )^{-1}\widetilde{{\mathcal {F}}m},$ see the definition below (6.2). More precisely,  for Schwartz functions $f_1, f_2$ we have

6.6 $$\begin{aligned}  &   T_m(f_1, f_2)(x) = -\frac{1}{2\pi } \int _{\mathbb {R}} \int _{\mathbb {R}} ({\mathcal {F}}m)(x - y, x- z) f_1(y) f_2(z) dy dz, \nonumber \\  &   \quad x \in {\mathbb {R}} {\setminus } ({{\,\textrm{supp}\,}}(f_1) \cap {{\,\textrm{supp}\,}}(f_2)). \end{aligned}$$

#### Proof

We have for $x \in {\mathbb {R}} {\setminus } ({{\,\textrm{supp}\,}}(f_1) \cap {{\,\textrm{supp}\,}}(f_2))$ that

$$\begin{aligned} T_m(f_1, f_2)(x)= &   \frac{1}{2\pi } \int _{{\mathbb {R}}} \int _{{\mathbb {R}}} m(\xi _1, \xi _2) ({\mathcal {F}} f_1)(\xi _1) ( {\mathcal {F}} f_2 )(\xi _2) e^{i (\xi _1 + \xi _2) x} d\xi _1 d\xi _2 \\= &   \frac{1}{2\pi } \int _{{\mathbb {R}}} \int _{{\mathbb {R}}} m(\xi _1, \xi _2) (({\mathcal {F}} f_1)(\xi _1) e^{i \xi _1 x} ) ( ( {\mathcal {F}} f_2 )(\xi _2) e^{i \xi _2 x}) d\xi _1 d\xi _2 \\= &   \frac{1}{2\pi } \int _{{\mathbb {R}}} \int _{{\mathbb {R}}} m(\xi _1, \xi _2) ({\mathcal {F}} f_1( \, \cdot \, + x))(\xi _1) ( {\mathcal {F}} f_2( \, \cdot \, + x) )(\xi _2) d\xi _1 d\xi _2 \\= &   \frac{1}{2\pi } \int _{{\mathbb {R}}} \int _{{\mathbb {R}}}({\mathcal {F}} m)(\xi _1, \xi _2) f_1( \xi _1 + x) f_2( \xi _2 + x) d\xi _1 d\xi _2 \\= &   \frac{1}{2\pi } \int _{{\mathbb {R}}} \int _{{\mathbb {R}}}({\mathcal {F}} m)(\xi _1-x, \xi _2-x) f_1( \xi _1) f_2( \xi _2 ) d\xi _1 d\xi _2. \\ \end{aligned}$$

As *m* is odd so is ${\mathcal {F}}m,$ hence we conclude (6.6).

To show that $T_m$ is indeed a Calderón–Zygmund operator as defined in Sect. 2.6, it remains to show conditions (6.1), (6.2), and boundedness of $T_m.$ The first two of these conditions hold by Proposition 6.2. Finally, the boundedness condition follows from [25, Theorem 8]. $\square$

#### Remark 6.4

For Calderón–Zygmund operators *T* on ${\mathbb {R}}$ with a convolution kernel

$$\begin{aligned} {\widetilde{K}}(x,y_1,\dotsc ,y_n)=K(x-y_1,\dotsc ,x-y_n), \quad x, y_1, \ldots , y_n \in {\mathbb {R}}, \end{aligned}$$

it holds that $\langle T(1,\dotsc ,1),\phi \rangle =0$ for all $\phi \in L_c^{\infty }({\mathbb {R}})$ with $\int _{{\mathbb {R}}} \phi dx = 0,$ i.e. $T(1,\dotsc ,1)$ vanishes in ${\textrm{BMO}}.$ As is common in the literature, we will refer to this as “$T(1,\dotsc ,1)=0$”. We decided to omit the detailed proof of this fact as it is commonly used in the literature. We refer the reader to the last equation in the proof of [20, Proposition 6] which applies to our situation; though we note that the proof there is only formal. Similarly, all partial adjoints $T^{*1}$ and $T^{*2}$ of *T* (defined via $\langle T^{*1} (f,g),h\rangle := \langle T(h,g),f\rangle ,$ $\langle T^{*2} (f,g),h\rangle := \langle T(f,h),g\rangle ,$ see [16]) vanish for these operators. See e.g. [16, 30] for well-defined constructions of these expressions. Hence in particular, for a bilinear Calderón–Zygmund operator with convolution kernel, it holds that $\langle T(1, 1),\phi \rangle =\langle T^{*1}(1, 1),\phi \rangle = \langle T^{*2}(1, 1),\phi \rangle =0$ for all $\phi \in L_c^{\infty }({\mathbb {R}})$ with $\int _{{\mathbb {R}}} \phi dx = 0.$

### Completely bounded estimates and constants for bilinear multipliers

The following is a special case of the main theorem of [15], specialised to our setting of Proposition 6.3 and Schatten classes. Unfortunately [15] does not keep track of the constants, though they can be made explicit by following the proof. We have outlined the proof of (6.8) in Appendix A. Note that Remark 6.4 implies the vanishing of the paraproduct terms in [15], which allows for a significantly better bound of (6.8) compared to general Calderón–Zygmund operators, see Remark 6.6.

#### Theorem 6.5

(Special case of [15, Theorem 1.1]) Let *T* be a bilinear Calderon–Zygmund operator on ${\mathbb {R}}.$ Then the bilinear operator

$$\begin{aligned} T_{cb}\Bigg (\sum _{j=1}^Jf_j\otimes y_j,\sum _{k=1}^Kg_k\otimes z_k\Bigg ):= \sum _{j,k}T(f_j,g_k)\otimes y_jz_k \end{aligned}$$

with $f_j,g_k\in L^{\infty }_c({\mathbb {R}}),$ $y_j\in S_{p_1},$ $z_k\in S_{p_2},$ extends to a bounded operator

$$\begin{aligned} T_{cb}:L^{p_1}({\mathbb {R}},S_{p_1})\times L^{p_2}({\mathbb {R}},S_{p_2})\rightarrow L^{p}({\mathbb {R}},S_{p}) \end{aligned}$$

for $p_1,p_2,p\in (1,\infty )$ such that $1/p_1+1/p_2=1/p.$ Moreover,  if for every $\phi \in L_c^{\infty }({\mathbb {R}})$ with $\int _{{\mathbb {R}}} \phi dx = 0,$ we have

6.7 $$\begin{aligned} \langle T(1,1), \phi \rangle = \langle T^{*1}(1,1), \phi \rangle = \langle T^{*2}(1,1), \phi \rangle = 0, \end{aligned}$$

then

6.8 $$\begin{aligned} \Vert T_{cb}:L^{p_1}({\mathbb {R}},S_{p_1})\times L^{p_2}({\mathbb {R}},S_{p_2})\rightarrow L^{p}({\mathbb {R}},S_{p}) \Vert \lesssim C(p, p_1, p_2), \end{aligned}$$

where

6.9 $$\begin{aligned}  &   C({p,p_1,p_2})= \beta _{p}\beta _{p_1}\beta _{p_2} +\min (\beta _{p_1}^2\beta _{p},\beta _{p}^2\beta _{p_1}) + \min (\beta _{p_2}^2\beta _{p},\beta _{p}^2\beta _{p_2})\nonumber \\  &   +\min (\beta _{p_2}^2\beta _{p_1},\beta _{p_1}^2\beta _{p_2}),\qquad \quad \end{aligned}$$

and $$\beta _q = q q^*,$$ $$1< q < \infty .$$

#### Remark 6.6

Without the condition (6.7) the paraproducts in the representation theorem described in Appendix A.1 do not vanish. Theorem 6.5 remains true but with a worse constant $C'(p,p_1, p_2)$ given by

$$\begin{aligned} C'(p,p_1, p_2)&= C(p,p_1, p_2) + \min (C''(p,p_1),C''(p,p_2)) \\&\quad + \min (C''(p_1,p_2),C''(p_1,p))+ \min (C''(p_2,p_1),C''(p_2,p)) , \\ C''(p,q)&= \beta _p^3\beta _q^2 C_{{\textrm{BMO}}_{q}}, \end{aligned}$$

where $C_{{\textrm{BMO}}_{p}}=2e(ep\Gamma (p))^{1/p}$ refers to the constant in the John–Nirenberg inequality, see e.g. [21]. For $p\rightarrow \infty ,$ we have $C_{{\textrm{BMO}}_{p}}=O(p).$ The constant $C'$ is derived through a combination of the permutation argument that we present at the end of Appendix A, and explicit calculations found in [38]. The facts we present in this remark shall not be used in this paper.

Next, we translate this statement to Fourier multipliers. This allows us to use transference to estimate bilinear Schur multipliers such as the ones in Proposition 3.4 by their corresponding Fourier multipliers.

#### Theorem 6.7

Let $m: {\mathbb {R}}^2 {\setminus } \{ 0 \} \rightarrow {\mathbb {R}}$ be smooth,  odd,  homogeneous,  and set $m(0,0) = 0.$ Then for $1<p,p_1, p_2 < \infty$ with $\frac{1}{p} = \frac{1}{p_1} + \frac{1}{p_2}$ we have

$$\begin{aligned} \Vert T_m: L^{p_1}({\mathbb {R}}^2, S_{p_1}) \times L^{p_2}({\mathbb {R}}^2, S_{p_2}) \rightarrow L^{p}({\mathbb {R}}^2, S_{p}) \Vert \lesssim C(p, p_1, p_2), \end{aligned}$$

where $C(p, p_1, p_2)$ is as in (6.9).

#### Proof

By Proposition 6.3, $T_m$ is a bilinear Calderón–Zygmund operator. By Remark 6.4, we see that (6.7) holds. Therefore, the statement follows directly from Theorem 6.5. $\square$

#### Theorem 6.8

Let $m: {\mathbb {R}}^2 {\setminus } \{ 0 \} \rightarrow {\mathbb {R}}$ be smooth,  odd,  homogeneous,  and set $m(0,0) = 0.$ Set

$$\begin{aligned} {\widetilde{m}}(\lambda _0, \lambda _1, \lambda _2) = m(\lambda _1 - \lambda _0, \lambda _2 - \lambda _1), \quad (\lambda _0, \lambda _1, \lambda _2) \in {\mathbb {R}}^3. \end{aligned}$$

Then

$$\begin{aligned}  &   \Vert M_{{\widetilde{m}}}: S_{p_1} \times S_{p_2} \rightarrow S_p \Vert \\  &   \quad \le \Vert T_m: L^{p_1}({\mathbb {R}}, S_{p_1}) \times L^{p_2}({\mathbb {R}}, S_{p_2}) \rightarrow L^{p}({\mathbb {R}}, S_{p}) \Vert \lesssim C(p, p_1, p_2), \end{aligned}$$

with $C(p, p_1, p_2)$ as given in (6.9).

#### Proof

We will apply [8, Theorem A] to a modification of *m* that is continuous at zero. Define $m(\lambda _1, \lambda _2; \mu _1, \mu _2) = m(\lambda _1 - \mu _1, \lambda _2 - \mu _2),$ $\lambda _i, \mu _i \in {\mathbb {R}}.$ Let $f \in C_b({\mathbb {R}})$ with compact support be such that $f \ge 0$ and $\Vert f \Vert _1 = 1.$ We set

$$\begin{aligned} m_f(\lambda _1, \lambda _2) = \int _{{\mathbb {R}}} \int _{{\mathbb {R}}} f(\mu _1) f(\mu _2) m(\lambda _1, \lambda _2; \mu _1, \mu _2) d \mu _1 d\mu _2, \end{aligned}$$

which is continuous. Set again ${\widetilde{m}}_f(\lambda _0, \lambda _1, \lambda _2) = m_f(\lambda _1 - \lambda _0, \lambda _2 - \lambda _1).$ It now follows from [8, Theorem A] that

$$\begin{aligned} \Vert M_{{\widetilde{m}}_f}: S_{p_1} \times S_{p_2} \rightarrow S_p \Vert \le \Vert T_{m_f}: L^{p_1}({\mathbb {R}}, S_{p_1}) \times L^{p_2}({\mathbb {R}}, S_{p_2}) \rightarrow L^{p}({\mathbb {R}}, S_{p}) \Vert . \end{aligned}$$

Next, observe that [9, Lemma 4.3] shows that $T_m$ and $T_{m(\, \cdot \,, \, \cdot \, ; \mu _1, \mu _2 )}$ have the same norm as bilinear maps. Therefore, it follows that

$$\begin{aligned}  &   \Vert T_{m_f}: L^{p_1}({\mathbb {R}}; S_{p_1}) \times L^{p_2}({\mathbb {R}}; S_{p_2}) \rightarrow L^{p}({\mathbb {R}}; S_{p}) \Vert \\  &   \quad \le \int _{{\mathbb {R}}} \int _{{\mathbb {R}}} f(\mu _1) f(\mu _2) \Vert T_{m}: L^{p_1}({\mathbb {R}}; S_{p_1}) \times L^{p_2}({\mathbb {R}}; S_{p_2}) \rightarrow L^{p}({\mathbb {R}}; S_{p}) \Vert d\mu _1 d\mu _2\\  &   \quad = \Vert T_{m}: L^{p_1}({\mathbb {R}}; S_{p_1}) \times L^{p_2}({\mathbb {R}}; S_{p_2}) \rightarrow L^{p}({\mathbb {R}}; S_{p}) \Vert . \end{aligned}$$

Combining the previous two estimates with Theorem 6.7 yields that

6.10 $$\begin{aligned} \Vert M_{{\widetilde{m}}_f}: S_{p_1} \times S_{p_2} \rightarrow S_p \Vert \lesssim C(p, p_1, p_2). \end{aligned}$$

Now replace *f* by functions $$f_j \in C_c({\mathbb {R}})$$ satisfying $$f_j \ge 0,$$ $$\Vert f_j \Vert _1 = 1,$$ $${{\,\textrm{supp}\,}}(f_j)\subset {{\,\textrm{supp}\,}}(f_{j-1}),$$ and $$\bigcap _j {{\,\textrm{supp}\,}}(f_j)=\{0\}.$$ Take $$x_1 \in S_{p_1} \cap S_2, x_2 \in S_{p_2}\cap S_2,$$ and $$x_3 \in S_{p^*} \cap S_2.$$ Assume that each of these operators is rank one with respective kernels $$A_i(s,t) = \xi _i(s) \eta _i(t)$$ (see Sect. 2.4), where we assume $$ \xi _i, \eta _i \in C_c({\mathbb {R}}),$$ $$ i=1,2,3.$$ Then as $$j \rightarrow \infty $$ we get for the Schatten class duality pairing

6.11 $$\begin{aligned}  &   \langle M_{ {\widetilde{m}}_{f_j} }(x_1, x_2), x_3 \rangle _{p, p^*} \nonumber \\  &   \quad =\int _{{\mathbb {R}}^3} {\widetilde{m}}_{f_j}(s_0, s_1, s_2) \xi _1(s_0) \xi _2(s_1) \xi _3(s_2) \eta _1(s_1) \eta _2(s_2) \eta _3(s_0) ds_0 ds_1 ds_2 \nonumber \\  &   \quad =\int _{{\mathbb {R}}^3} \int _{{\mathbb {R}}^2} f_j(\mu _1) f_j(\mu _2) m(s_1 - s_0 - \mu _1, s_2 - s_1 - \mu _2) \nonumber \\  &   \times \xi _1(s_0) \xi _2(s_1) \xi _3(s_2) \eta _1(s_1) \eta _2(s_2) \eta _3(s_0) d\mu _1 d\mu _2 ds_0 ds_1 ds_2 \nonumber \\  &   \quad =\int _{{\mathbb {R}}^3} \int _{{\mathbb {R}}^2} f_j(\mu _1) f_j(\mu _2) m(s_1 - s_0 , s_2 - s_1 )\times \xi _1(s_0 - \mu _1) \xi _2(s_1) \xi _3(s_2 \nonumber \\  &   +\mu _2) \eta _1(s_1) \eta _2(s_2 + \mu _2) \eta _3(s_0 - \mu _1) d\mu _1 d\mu _2 ds_0 ds_1 ds_2. \end{aligned}$$

As each $\xi _j$ and $\eta _j$ is continuous and compactly supported, we have

6.12 $$\begin{aligned}  &   \int _{{\mathbb {R}}^2} f_j(\mu _1) f_j(\mu _2) \xi _1(s_0 - \mu _1) \xi _2(s_1) \xi _3(s_2 +\mu _2) \eta _1(s_1) \eta _2(s_2 + \mu _2) \eta _3(s_0 - \mu _1) d\mu _1 d\mu _2 \nonumber \\  &   \quad \xrightarrow {j\rightarrow \infty } \;\xi _1(s_0 ) \xi _2(s_1) \xi _3(s_2) \eta _1(s_1) \eta _2(s_2) \eta _3(s_0 ), \end{aligned}$$

in the $L^1({\mathbb {R}}^3)$-norm, where we see the expressions in (6.12) as functions of $(s_0, s_1, s_2) \in {\mathbb {R}}^3.$ Therefore, taking the limit $j \rightarrow \infty$ in (6.11) gives

6.13 $$\begin{aligned}  &   \langle M_{ {\widetilde{m}}_{f_j} }(x_1, x_2), x_3 \rangle _{p, p^*} \nonumber \\  &   \quad \xrightarrow {j\rightarrow \infty } \int _{{\mathbb {R}}^3} m(s_1 - s_0 , s_2 - s_1 ) \xi _1(s_0 ) \xi _2(s_1) \xi _3(s_2) \eta _1(s_1) \eta _2(s_2) \eta _3(s_0) ds_0 ds_1 ds_2 \nonumber \\  &   \quad = \langle M_{ {\widetilde{m}} }(x_1, x_2), x_3 \rangle _{p, p^*} \nonumber \\ \end{aligned}$$

By linearity, density, and uniform boundedness of $M_{{\widetilde{m}}}$ and $M_{{\widetilde{m}}_{f_j}}$ as maps $S_2 \times S_2 \rightarrow S_2$ (see Sect. 2.4) the convergence (6.13) holds for any $x_1 \in S_2 \cap S_{p_1}, x_2 \in S_2 \cap S_{p_2}, x_3 \in S_2 \cap S_{p^*}.$ Hence,

$$\begin{aligned}  &   \Vert M_{{\widetilde{m}}}: S_{p_1} \times S_{p_2} \rightarrow S_p \Vert \\  &   \quad = \sup _{\begin{array}{c} x_1 \in S_2 \cap S_{p_1}, x_2 \in S_2 \cap S_{p_2}, x_3 \in S_2 \cap S_{p^*}, \\ \Vert x_1 \Vert _{p_1} = \Vert x_2 \Vert _{p_2} = \Vert x_3 \Vert _{p^*} = 1 \end{array}} \vert \langle M_{ {\widetilde{m}} }(x_1, x_2), x_3 \rangle _{p, p^*} \vert \\  &   \quad = \sup _{\begin{array}{c} x_1 \in S_2 \cap S_{p_1}, x_2 \in S_2 \cap S_{p_2}, x_3 \in S_2 \cap S_{p^*}, \\ \Vert x_1 \Vert _{p_1} = \Vert x_2 \Vert _{p_2} = \Vert x_3 \Vert _{p^*} = 1 \end{array}} \lim _{j \rightarrow \infty } \vert \langle M_{ {\widetilde{m}}_{f_j} }(x_1, x_2), x_3 \rangle _{p, p^*} \vert \\  &   \quad \le \limsup _{j \rightarrow \infty } \Vert M_{{\widetilde{m}}_{f_j}}: S_{p_1} \times S_{p_2} \rightarrow S_p \Vert , \end{aligned}$$

which concludes the proof. $\square$

## Proof of Theorem A and extrapolation

### Main result

We now collect all estimates we have obtained so far in this paper.

#### Theorem 7.1

(Theorem A) For every $f \in C^2({\mathbb {R}})$ and for every $1<p, p_1, p_2 < \infty$ such that $\frac{1}{p} = \frac{1}{p_1} + \frac{1}{p_2}$ we have that

$$\begin{aligned} \Vert M_{f^{[2]}}: S_{p_1} \times S_{p_2} \rightarrow S_{p} \Vert \lesssim D(p, p_1, p_2) \Vert f'' \Vert _\infty , \end{aligned}$$

where

$$\begin{aligned} D(p, p_1, p_2) = C(p, p_1, p_2) ( \beta _{p_1} + \beta _{p_2} ) + \beta _{p_1} \beta _{p_2} (\beta _p + \beta _{p_1} + \beta _{p_2} ) \end{aligned}$$

where $C(p, p_1,p_2)$ was defined in (6.9) and $\beta _q = q q^*.$

#### Proof

Consider the decomposition of $M_{f^{[2]}}$ given in (3.8) in terms of bilinear Schur multipliers of Toeplitz form and linear Schur multipliers. It is sufficient to show that each of these maps are bounded on the corresponding Schatten classes. Each of the functions $a_1 := \epsilon _1 {\widetilde{\theta }}_1 \psi _1,$ $a_2:= \epsilon _2 {\widetilde{\theta }}_1 (1 - \psi _1),$ $a_3 := \epsilon _3 {\widetilde{\theta }}_2 \psi _2,$ $a_4 := \epsilon _1 {\widetilde{\theta }}_2 (1 - \psi _2),$ $a_5 := \epsilon _2 {\widetilde{\theta }}_2 \psi _2$ and $a_6 := \epsilon _3 {\widetilde{\theta }}_3(1- \psi _3)$ is smooth, odd, homogeneous, and has value zero at zero. Note that we added the $\epsilon _i$ terms to assure that the functions are odd. Therefore by Theorem 6.8 we get the bounds

$$\begin{aligned} \Vert M_{a_i} : S_{p_1} \times S_{p_2} \rightarrow S_{p} \Vert \lesssim C(p, p_1, p_2), \quad 1 \le i \le 6. \end{aligned}$$

We shall only use this fact for $i=1,2.$ By Corollary 5.2 we also get

$$\begin{aligned} \Vert M_{a_i} : S_{p_1} \times S_{p_2} \rightarrow S_{p} \Vert \lesssim p_1p_1^*p_2 p_2^*= \beta _{p_1} \beta _{p_2}, \quad 3 \le i \le 6. \end{aligned}$$

For the linear term $M_{\epsilon \phi _f}$ , we apply Remark 4.5 to see that for any $1< q < \infty ,$

$$\begin{aligned} \Vert M_{\epsilon \phi _f}: S_{q} \rightarrow S_{q} \Vert \lesssim \Vert f'' \Vert _{\infty } q q^*= \beta _q, \end{aligned}$$

and similarly for $\epsilon \mathring{\phi }_f.$ These estimates together with the decomposition (3.8) allow us to conclude

$$\begin{aligned} \Vert M_{f^{[2]}}: S_{p_1} \times S_{p_2} \rightarrow S_{p} \Vert \lesssim C(p, p_1, p_2) ( \beta _{p_1} + \beta _{p_2} ) + \beta _{p_1} \beta _{p_2} (\beta _p + \beta _{p_1} + \beta _{p_2} ). \end{aligned}$$

$$\square $$

#### Remark 7.2

We examine the constant *D*(*p*, 2*p*, 2*p*) with $1< p < \infty$ and its asymptotics for *p* going either to $\infty$ or 1. Note that if $p \searrow 1$ then $(2p)^*\nearrow 2.$ In fact, $(2p)^*$ is uniformly bounded for $1< p < \infty .$ We therefore find for $1< p < \infty$ that

$$\begin{aligned} D(p, 2p, 2p) \approx p^4 p^*. \end{aligned}$$

#### Remark 7.3

The *p*-dependence of the norm of the triple operator integral appearing in [35, Remark 5.4] is not made explicit in [35]. Following the proof of [35] in the bilinear case we find that $D(p, 2p, 2p) = O( p^{12})$ as $p\rightarrow \infty .$ This is justified as follows.

1. The three triangular truncations used on [35, p. 533] yield a factor of order $O(p^3).$
2. Estimating the linear terms in decomposition [35, Eqn. (4.3)] yields a factor of order $O(p^3),$ arising from the application of [35, Lemma 4.5], which is of order $O(p^3).$
3. Estimating the bilinear terms in decomposition [35, Eqn. (4.5)] yields a factor of order $O(p^6).$ As shown on [35, p. 519], these estimates require two applications of [35, Lemma 4.5], which is of order $O(p^3),$ to estimate the operator $R_s$ of [35].

A detailed account of these facts is contained in [38]. Our proof thus gives a significant improvement of estimate for *D*(*p*, 2*p*, 2*p*) from $O( p^{12})$ to $O(p^4)$ in case $p \rightarrow \infty .$ In Sect. 8 we show that the order of *D*(*p*, 2*p*, 2*p*) is at least $O(p^2)$ for $p \rightarrow \infty .$

### Extrapolation

Let $x \in B(H)$ be a compact operator. We set the decreasing rearrangement of $t \in [0, \infty )$ as

$$\begin{aligned} \mu _t(x) = \inf \{ \Vert x p \Vert \mid p \in B(H) \text { projection with } \textrm{Tr}(p) \le t \}. \end{aligned}$$

We define $M_{1, \infty }$ as the Marcinkiewicz space of all compact operators *x* such that

$$\begin{aligned} \Vert x \Vert _{M_{1, \infty }} := \sup _{t \in [0, \infty )} \log (1+t)^{-1} \int _0^t \mu _s(x) ds < \infty . \end{aligned}$$

Theorem 7.1 now yields the following extrapolation result, which should be compared to [2, Corollary 5.6].

#### Theorem 7.4

For every $f \in C^2({\mathbb {R}})$ we have

$$\begin{aligned} \Vert M_{f^{[2]}}: S_{2} \times S_{2} \rightarrow M_{1, \infty } \Vert < \infty . \end{aligned}$$

#### Proof

Let $s > 0$ be large, set $p = \log (s)$ and set $q = p^*= p (p-1)^{-1}$ to be the Hölder conjugate of *p*. Note that as $s \rightarrow \infty$ we thus have $q \searrow 1.$ Let $x,y \in S_{2}$ and set $T = M_{f^{[2]}}(x,y).$ Then by Hölder’s inequality, Theorem 7.1, and the fact that the embedding $S_{2 }\hookrightarrow S_{2q}$ is contractive, we have

$$\begin{aligned} \int _0^s \mu _t(T) dt \le s^{\frac{1}{p}} \left( \int _0^s \mu _t(T)^q dt \right) ^{\frac{1}{q}} \le s^{\frac{1}{p}} \Vert T \Vert _q\lesssim &   s^{\frac{1}{p}} D(q, 2q, 2q) \Vert x \Vert _{2q} \Vert y \Vert _{2q}\\\le &   s^{\frac{1}{p}} D(q, 2q, 2q) \Vert x \Vert _{2} \Vert y \Vert _{2}. \end{aligned}$$

We have

$$\begin{aligned} s^{\frac{1}{p}} D(q, 2q, 2q) \le 100 s^{\frac{1}{p}} q^*= 100 e^{\frac{1}{p} \log (s)} p = 100 e^1 \log (s). \end{aligned}$$

So we see that

$$\begin{aligned} \int _0^s \mu _t(T) dt \lesssim \log (s) \Vert x \Vert _{2} \Vert y \Vert _{2}. \end{aligned}$$

This proves the extrapolation result. $\square$

#### Remark 7.5

The question what the best recipient space for triple operator integrals of second order divided difference functions is remains open. In particular we do not know whether for $f \in C^2({\mathbb {R}})$ we have

$$\begin{aligned} \Vert M_{f^{[2]}}: S_{2} \times S_{2} \rightarrow S_{1, \infty } \Vert < \infty , \end{aligned}$$

where $S_{1, \infty }$ is the weak $S_1$-space. Only in case $f(s) = s \vert s \vert ,$ as well as some simple modifications of this function, this question is answered in the affirmative [7]. In Sect. 8 we prove lower bounds for Schur multipliers associated with the latter function.

## Lower bounds and proof of Theorem B

In this section we investigate the lower bounds of Schur multipliers of second order divided difference functions. In [11] it was already shown that for general $f \in C^2({\mathbb {R}})$ we do not necessarily have that $M_{f^{[2]}}$ maps $S_2 \times S_2$ to $S_1.$ The counterexample of [11] is given by the function $f(s) = s \vert s\vert , s \in {\mathbb {R}}$ (or in fact a perturbation of this function around zero that makes the function $C^2$). Here we improve on this result by providing explicit lower bounds for the corresponding problem on Schatten classes. Our proof gives in fact better asymptotics for $p \rightarrow \infty$ than [11], as we explain in Remark 8.5.

### Theorem 8.1

(Theorem B, Part 1) Let $f(s) = s \vert s\vert , s \in {\mathbb {R}}.$ Then for every $1< p < \infty$ we have

$$\begin{aligned} \Vert M_{f^{[2]}}: S_{2p} \times S_{2p} \rightarrow S_{p} \Vert \gtrsim p^2. \end{aligned}$$

We prove Theorem 8.1 through a couple of lemmas.

### Lemma 8.2

Let $f(s) = s \vert s\vert , s \in {\mathbb {R}}.$ Let $q \in (0,1)$ and let $i,j,l \in {\mathbb {N}}$ be such that $i \not = j$ and $j \not = l.$ Then

8.1 $$\begin{aligned} \lim _{k \rightarrow \infty } f^{[2]}(q^{ki}, -q^{kj}, q^{kl}) = \left\{ \begin{array}{ll} -1 &  \quad {\text {if }} j< i, {\text { and }} j < l, \\ 1 &  {\text {otherwise}}. \end{array} \right. \end{aligned}$$

### Proof

Let $\lambda _0, \lambda _2 > 0$ and $\lambda _1 < 0,$ then $f(\lambda _0) = \lambda _0^2, f(\lambda _1) = - \lambda _1^2, f(\lambda _2) = \lambda _2^2.$ First expand

8.2 $$\begin{aligned} f^{[2]}(\lambda _0, \lambda _1, \lambda _2)= &   \frac{ f^{[1]}(\lambda _0, \lambda _1) - f^{[1]}(\lambda _1, \lambda _2) }{ \lambda _0 - \lambda _2}\nonumber \\= &   \frac{1}{\lambda _0 - \lambda _2} \left( \frac{f(\lambda _0) - f(\lambda _1)}{ \lambda _0 - \lambda _1} - \frac{f(\lambda _1) - f(\lambda _2)}{ \lambda _1 - \lambda _2} \right) \nonumber \\= &   \frac{1}{\lambda _0 - \lambda _2} \left( \frac{ \lambda _0^2 + \lambda _1^2}{ \lambda _0 - \lambda _1} - \frac{ - \lambda _1^2 - \lambda _2^2}{ \lambda _1 - \lambda _2} \right) . \end{aligned}$$

We set ${\widetilde{\lambda }}_1 := -\lambda _1.$ Then $\lambda _0, {\widetilde{\lambda }}_1, \lambda _2 >0$ and

8.3 $$\begin{aligned} f^{[2]}(\lambda _0, -{\widetilde{\lambda }}_1, \lambda _2)= &   \frac{(\lambda _0^2 + {\widetilde{\lambda }}_1^2)({\widetilde{\lambda }}_1 + \lambda _2) - ({\widetilde{\lambda }}_1^2 + \lambda _2^2)(\lambda _0 + {\widetilde{\lambda }}_1) }{(\lambda _0 - \lambda _2)(\lambda _0 + {\widetilde{\lambda }}_1) ({\widetilde{\lambda }}_1 + \lambda _2) } \nonumber \\= &   \frac{\lambda _0^2 {\widetilde{\lambda }}_1 + \lambda _0^2 \lambda _2 + {\widetilde{\lambda }}_1^2 \lambda _2 - {\widetilde{\lambda }}_1^2 \lambda _0 - \lambda _2^2 \lambda _0 - \lambda _2^ 2 {\widetilde{\lambda }}_1 }{(\lambda _0 - \lambda _2)(\lambda _0 + {\widetilde{\lambda }}_1) ({\widetilde{\lambda }}_1 + \lambda _2) } \nonumber \\= &   \frac{ (\lambda _0 + \lambda _2){\widetilde{\lambda }}_1 + \lambda _0 \lambda _2 - {\widetilde{\lambda }}_1^2 }{ (\lambda _0 + {\widetilde{\lambda }}_1) ({\widetilde{\lambda }}_1 + \lambda _2) }. \end{aligned}$$

Let $q \in (0,1)$ as in the statement of the lemma and let $k \in {\mathbb {N}}.$ Set $\lambda _0 = q^{ki}, {\widetilde{\lambda }}_1 = q^{kj}, \lambda _2 = q^{kl},$ where $i,j,l \in {\mathbb {N}}$ are natural numbers with $i \not = j$ and $j \not = l.$ By considering each of the 6 possible orderings of $\lambda _0, {\widetilde{\lambda }}_1,$ and $\lambda _2,$ we see from (8.3) that

8.4 $$\begin{aligned} \lim _{k \rightarrow \infty } f^{[2]}(q^{ki}, -q^{kj}, q^{kl}) = \left\{ \begin{array}{ll} -1 &  \quad {\text {if }} j< i {\text { and }} j < l. \\ 1 & \quad {\text {otherwise}}. \end{array} \right. \end{aligned}$$

This concludes the proof. $\square$

### Lemma 8.3

Let $f(s) = s \vert s\vert , s \in {\mathbb {R}}.$ Let $q \in (0,1)$ and for $i,j,l \in {\mathbb {N}},$ let ${\phi _k(i,j,l) := (q^{ki}, -q^{kj}, q^{kl})}.$ Then for all $1< p < \infty$ we have

$$\begin{aligned} \Vert M_{f^{[2]} \circ \phi _k}: S_{2p}( \ell ^2({\mathbb {N}}) ) \times S_{2p}(\ell ^2({\mathbb {N}}) ) \rightarrow S_{p}(\ell ^2({\mathbb {N}})) \Vert \le \Vert M_{f^{[2]}}: S_{2p} \times S_{2p} \rightarrow S_{p} \Vert . \end{aligned}$$

### Proof

Let $F, G \subseteq {\mathbb {R}}$ be finite sets not containing 0. Then $F \cup G$ is contained in a set $X_\delta \subseteq {\mathbb {R}}$ of the form $(- \infty , -\delta ) \cup (\delta , \infty )$ for some $\delta > 0.$ Note that $f^{[2]}$ is continuous on $X_\delta \times X_\delta \times X_\delta$ and so we may apply [8, Theorem 2.2]. By using respectively a restriction of the domain of a bilinear Schur multiplier, then applying [8, Theorem 2.2] and then again a restriction of the domain, we get

$$\begin{aligned}  &   \Vert M_{f^{[2]}}: S_{2p}(\ell ^2(F), \ell ^2(G)) \times S_{2p}(\ell ^2(G), \ell ^2(F)) \rightarrow S_{p}(\ell ^2(F)) \Vert \\  &   \quad \le \Vert M_{f^{[2]}}: S_{2p}(\ell ^2(F \cup G)) \times S_{2p}(\ell ^2(F \cup G)) \rightarrow S_{p}(\ell ^2(F \cup G)) \Vert \\  &   \quad \le \Vert M_{f^{[2]}}: S_{2p}(L^2(X_\delta )) \times S_{2p}(L^2(X_\delta )) \rightarrow S_{p}(L^2(X_\delta )) \Vert \\  &   \quad \le \Vert M_{f^{[2]}}: S_{2p}(L^2({\mathbb {R}})) \times S_{2p}(L^2({\mathbb {R}})) \rightarrow S_{p}(L^2({\mathbb {R}})) \Vert . \end{aligned}$$

Now let $F, G \subseteq {\mathbb {R}}$ be any subsets not containing 0. The union of all vector spaces $S_{p}(\ell ^2(F_0), \ell ^2(G_0))$ with $F_0 \subseteq F, G_0\subseteq G$ finite is dense in $S_{p}(\ell ^2(F), \ell ^2(G)).$ Therefore we have,

$$\begin{aligned}  &   \Vert M_{f^{[2]}}: S_{2p}(\ell ^2(F), \ell ^2(G)) \times S_{2p}(\ell ^2(G), \ell ^2(F)) \rightarrow S_{p}(\ell ^2(F)) \Vert \\  &   \quad = \sup _{F_0 \subseteq F, G_0 \subseteq G \text { finite}} \Vert M_{f^{[2]}}: S_{2p}(\ell ^2(F_0), \ell ^2(G_0))\\  &   \qquad \qquad \qquad \times S_{2p}(\ell ^2(G_0), \ell ^2(F_0)) \rightarrow S_{p}(\ell ^2(F_0)) \Vert \\  &   \quad \le \Vert M_{f^{[2]}}: S_{2p}(L^2({\mathbb {R}})) \times S_{2p}(L^2({\mathbb {R}})) \rightarrow S_{p}(L^2({\mathbb {R}})) \Vert . \end{aligned}$$

Now let

$$\begin{aligned} F_k = \{ q^{k i } \mid i \in {\mathbb {N}} \}, \quad G_k = - F_k = \{ - q^{k i } \mid i \in {\mathbb {N}} \}. \end{aligned}$$

Let $\delta _x$ be as in Sect. 2.4 and define unitary maps

$$\begin{aligned} U_k: \ell ^2({\mathbb {N}}) \rightarrow \ell ^2(F_k): \delta _n \mapsto \delta _{q^{kn}}, \quad V_k: \ell ^2( {\mathbb {N}} ) \rightarrow \ell ^2( G_k): \delta _n \mapsto \delta _{-q^{kn}}. \end{aligned}$$

By interpreting these maps as base change operators, one can relate $M_{f^{[2]}\circ \phi _k}$ and $M_{f^{[2]}}$ via

$$\begin{aligned} M_{f^{[2]} \circ \phi _k}( x, y ) = U_k^*M_{f^{[2]}} ( U_k x V_k^*, V_k y U_k ) U_k^*, \quad x,y \in S_2(\ell ^2({\mathbb {N}})). \end{aligned}$$

Therefore

$$\begin{aligned}  &   \Vert M_{f^{[2]} \circ \phi _k}: S_{2p}(\ell ^2({\mathbb {N}}) ) \times S_{2p}(\ell ^2({\mathbb {N}}) ) \rightarrow S_{p}(\ell ^2({\mathbb {N}})) \Vert \\  &   \quad = \Vert M_{f^{[2]} }: S_{2p}(\ell ^2(F_k), \ell ^2(G_k)) \times S_{2p}(\ell ^2(G_k), \ell ^2( F_k ) ) \rightarrow S_{p}(F_k) \Vert \\  &   \quad \le \Vert M_{f^{[2]}}: S_{2p}(L^2({\mathbb {R}})) \times S_{2p}(L^2({\mathbb {R}})) \rightarrow S_{p}(L^2({\mathbb {R}})) \Vert . \end{aligned}$$

This concludes the proof. $\square$

### Proof of Theorem 8.1

Let $T^\pm = T_{\widetilde{h_{\pm }}}: S_{2p}(\ell ^2({\mathbb {N}})) \rightarrow S_{2p}(\ell ^2({\mathbb {N}}))$ be the triangular truncation given by the Schur multiplier with symbol,

$$\begin{aligned} \widetilde{h_\pm }(\lambda , \mu ) = h_\pm (\lambda - \mu ), \quad h_\pm (\lambda ) = \left\{ \begin{array}{ll} 1 &  \quad \text {if } \pm \lambda < 0, \\ 0 &  \quad \text {if } \pm \lambda \ge 0, \end{array} \right. \end{aligned}$$

There exist constants $C, D >0$ such that for all $1<p<\infty ,$

8.5 $$\begin{aligned} C p< \Vert T^{\pm }: S_{2p}( \ell ^2({\mathbb {N}}) ) \rightarrow S_{2p}( \ell ^2({\mathbb {N}}) ) \Vert < D p. \end{aligned}$$

The lower bound of this inequality, which is well-known and most relevant to us, follows for instance from the explicit sequence of singular values of the Volterra operator due to Krein (see [19, Theorem IV.8.2 and IV.7.4]). Now set $M^+ = T^+ - T^-$ and $M^- = T^- - T^+.$ Let *P* be the projection of $S_{2p}(\ell ^2({\mathbb {N}}))$ onto the diagonal elements. Then *P* is a contraction (see [4, Lemma 2.1]). Note that $M^\pm$ is a Schur multiplier acting on $S_{2p}( \ell ^2({\mathbb {N}}) )$ with symbol ${\widetilde{H}}_{\pm }(\lambda , \mu ) = H_\pm (\lambda - \mu ),$ $\lambda , \mu \in {\mathbb {N}},$ and $H_{\pm }(\lambda ) = \pm 1$ if $\pm \lambda < 0.$ Similarly, *P* is a Schur multiplier with symbol $p(\lambda , \mu ) = 1$ if $\lambda = \mu$ and $p(\lambda , \mu ) = 0$ otherwise, where again $\lambda , \mu \in {\mathbb {N}}.$ In particular, $T^+ = \frac{1}{2} (M^+ + \textrm{Id} - P).$ Therefore, from (8.5) we get by the reverse triangle inequality

$$\begin{aligned} 2C p - 2 < \Vert (M^+ + \textrm{Id} - P) \Vert - \Vert \textrm{Id} - P \Vert \le \Vert M^+ \Vert , \end{aligned}$$

where all norms are operator norms of linear maps on $S_{2p}( \ell ^2({\mathbb {N}}) ).$ For $1< p < \infty$ we have by (2.2), which also hold for discrete symbols,

$$\begin{aligned}  &   1 = \Vert {\widetilde{H}}_{+} \Vert _{\ell ^\infty ({\mathbb {N}} \times {\mathbb {N}})} = \Vert M^+: S_{2}( \ell ^2({\mathbb {N}}) ) \rightarrow S_{2}( \ell ^2({\mathbb {N}}) )\Vert \\  &   \le \Vert M^+: S_{2p}( \ell ^2({\mathbb {N}}) ) \rightarrow S_{2p}( \ell ^2({\mathbb {N}}) )\Vert . \end{aligned}$$

We thus obtain $\max ( 2Cp - 2, 1) \le \Vert M^+: S_{2p}( \ell ^2({\mathbb {N}}) ) \rightarrow S_{2p}( \ell ^2({\mathbb {N}}) ) \Vert .$ Furthermore, for any ${1< p < \infty }$ we have $\frac{2}{3} C p < \max ( 2Cp - 2, 1) .$ Altogether, we see that for all $1< p < \infty$ we have

8.6 $$\begin{aligned} \frac{2}{3} C p < \Vert M^{+}: S_{2p}( \ell ^2({\mathbb {N}}) ) \rightarrow S_{2p}( \ell ^2({\mathbb {N}}) ) \Vert . \end{aligned}$$

Now fix $1< p < \infty .$ By (8.6) we can for any $\epsilon > 0$ choose $x \in S_{2p}(\ell ^2({\mathbb {N}}))$ such that

8.7 $$\begin{aligned} \Vert M^+(x) \Vert _{2p} > \frac{2}{3} C p (\Vert x \Vert _{2p} - \epsilon ). \end{aligned}$$

It follows that

$$\begin{aligned} \Vert M^-( x^*) M^+( x )\Vert _p = \Vert M^+(x)^*M^+(x)\Vert _p = \Vert M^+(x)\Vert _{2p}^2 > \frac{4}{9} C^2 p^2 (\Vert x \Vert _{2p} - \epsilon )^2. \end{aligned}$$

Now for $i,j,l \in {\mathbb {N}}$ as in Lemma 8.3 we define $\phi _k(i,j,l) := (q^{ki}, -q^{kj}, q^{kl}).$ Then the limit in Lemma 8.2 shows that for $x,y \in S_{2p}(\ell ^2(F))$ with $F \subseteq {\mathbb {N}}$ finite we have

8.8 $$\begin{aligned}  &   \lim _k M_{f^{[2]} \circ \phi _k}((1-P)(y),(1-P)(x)) \nonumber \\  &   \quad = \lim _k \!\!\!\!\!\!\!\! \sum _{\begin{array}{c} \lambda _0, \lambda _1, \lambda _2 \in F,\\ \lambda _0 \not = \lambda _1, \lambda _1 \not = \lambda _2 \end{array}} \!\!\!\! (f^{[2]} \circ \phi _k)(\lambda _0, \lambda _1, \lambda _2) p_{\lambda _0} x p_{\lambda _1} y p_{\lambda _2} \nonumber \\  &   \quad = M^-((1-P)(y)) M^+((1-P)(x)) \nonumber \\  &   \quad = M^-(y) M^+(x). \end{aligned}$$

As we are deal with finite dimensional spaces, the limit (8.8) holds in the norm of $S_{p}(\ell ^2(F)).$ Moreover, as $M_{f^{[2]} \circ \phi _k}$ is bounded uniformly in *k* by Lemma 8.3, it follows by density of the span of $\{ S_{2p}(\ell ^2(F)) \mid F \subseteq {\mathbb {N}} \text { finite} \}$ in $S_{2p}(\ell ^2({\mathbb {N}}))$ that this convergence holds for any $x,y \in S_{2p}(\ell ^2({\mathbb {N}})).$

We now have the estimates

$$\begin{aligned}  &   \frac{4}{9} C^2 p ^2 (\Vert x \Vert _{2p} - \epsilon )^2 < \Vert M^-( x^*) M^+( x ) \Vert _p \\  &   \quad \le \limsup _k \Vert M_{f^{[2]} \circ \phi _k}((1-P)(x)^*, (1-P)(x) ) \Vert _{p}. \end{aligned}$$

Thus by Lemma 8.3 we get,

$$\begin{aligned}  &   \frac{4}{9} C^2 p ^2 (\Vert x \Vert _{2p} - \epsilon )^2 < \Vert M_{f^{[2]}}: S_{2p} \times S_{2p} \rightarrow S_p \Vert \Vert (1-P)(x) \Vert _{2p}^2 \\  &   \quad \le 4 \Vert M_{f^{[2]}}: S_{2p} \times S_{2p} \rightarrow S_p \Vert \Vert x \Vert _{2p}^2. \end{aligned}$$

Hence

$$\begin{aligned} \Vert M_{f^{[2]}}: S_{2p} \times S_{2p} \rightarrow S_{p} \Vert \gtrsim p^2. \end{aligned}$$

$$\square $$

Note that if $p \searrow 1,$ then $2p \searrow 2$ and hence the norm in (8.6) remains bounded. Therefore, we need a different proof to treat $p \searrow 1,$ which we present below as a separate theorem. Since many parts of the proof are similar to the proof of Theorem 8.1 we present it in a more concise manner.

### Theorem 8.4

(Theorem B, Part 2) Let $f(s) = s \vert s\vert , s \in {\mathbb {R}}.$ Then for every $1< p < \infty$ we have

$$\begin{aligned} \Vert M_{f^{[2]}}: S_{2p} \times S_{2p} \rightarrow S_{p} \Vert \gtrsim p^*. \end{aligned}$$

### Proof

Assume that $\lambda _0 >0,$ $\lambda _1 \ge 0,$ $\lambda _2 < 0$ so that $f(\lambda _0) = \lambda _0^2,$ $f(\lambda _1) = \lambda _1^2,$ $f(\lambda _2) = -\lambda _2^2.$ In the proof we will take $\lambda _1$ to be very close to zero and infinitesimally smaller than both $\lambda _0$ and $\vert \lambda _2 \vert .$ As in (8.2), we expand

8.9 $$\begin{aligned} f^{[2]}(\lambda _0, \lambda _1, \lambda _2) = \frac{1}{\lambda _0 - \lambda _2} \left( \frac{ \lambda _0^2 - \lambda _1^2 }{ \lambda _0 - \lambda _1} - \frac{ \lambda _1^2 + \lambda _2^2}{ \lambda _1 - \lambda _2} \right) . \end{aligned}$$

Take some $q \in (0,1)$ fixed and let $k \in {\mathbb {N}}.$ Assume that $\lambda _0 = \lambda _0(k) = q^{ki}, \lambda _1 = q^{k(i+l)}, \lambda _2 = \lambda _2(k) = - q^{kl}$ for $i, l \in {\mathbb {N}}$ different natural numbers. By our definition zero is not included in ${\mathbb {N}},$ and therefore $\lambda _1$ is strictly smaller than both $\lambda _0$ and $\vert \lambda _2\vert .$

Again we see from (8.9) that

8.10 $$\begin{aligned} \lim _{k \rightarrow \infty } f^{[2]}(q^{ki}, q^{k(i+l)}, -q^{kl}) = \left\{ \begin{array}{ll} 1 &  \quad \text {if } i< l, \\ -1 &  \quad \text {if } l < i. \end{array} \right. \end{aligned}$$

Now for $i, j, l \in {\mathbb {N}},$ let

$$\begin{aligned} \phi _k(i,j,l) = (q^{ki}, q^{k(i+l)}, -q^{kl}). \end{aligned}$$

Let the diagonal projection *P* and the Schur multiplier $M^+$ be defined as in the proof of Theorem 8.1. Then from the limit (8.10) and the fact that as in the proof of Theorem 8.1 we can show that $M_{f^{[2]} \circ \phi _k}$ is bounded uniformly in *k*,  we can show that

$$\begin{aligned} M^+ (y x) = \lim _k (1-P)( M_{f^{[2]} \circ \phi _k}( y ,x) ), \quad y, x \in S_{2p}({\mathbb {N}}), \end{aligned}$$

with convergence in the norm of $S_{p}({\mathbb {N}}).$

We recall from (8.6) and by duality, that there exist $C, D >0$ such that for every $1< p < \infty$ we have

8.11 $$\begin{aligned} C p p^*< \Vert M^+: S_{p}({\mathbb {N}} ) \rightarrow S_{p}({\mathbb {N}} ) \Vert < D p p^*. \end{aligned}$$

For any $\epsilon > 0$ and $1< p < \infty$ we can choose $z \in S_{p}({\mathbb {N}} )$ such that

$$\begin{aligned} \Vert M^+(z) \Vert _{p} > C p (\Vert z \Vert _{p} - \epsilon ). \end{aligned}$$

Write $z = y x$ with $y,x \in S_{2p}({\mathbb {N}} )$ such that $\Vert z \Vert _p = \Vert y \Vert _{2p} \Vert x \Vert _{2p}.$ We now have the estimates

$$\begin{aligned}  &   C p^*(\Vert z \Vert _{p} - \epsilon ) < \Vert M^+( y , x) \Vert _p \\  &   \quad \le \limsup _k \Vert (1-P)( M_{f^{[2]} \circ \phi _k}( x, y ) ) \Vert _{p} \\  &   \quad \le \limsup _k \Vert M_{f^{[2]} \circ \phi _k}( x, y ) \Vert _{p}. \end{aligned}$$

Then by [8, Theorem 2.2],

$$\begin{aligned}  &   C p^*(\Vert z \Vert _{p} - \epsilon ) < \Vert M_{f^{[2]}}: S_{2p} \times S_{2p} \rightarrow S_p \Vert \Vert x \Vert _{2p}\Vert y \Vert _{2p} \ \\  &   \quad \le \Vert M_{f^{[2]}}: S_{2p} \times S_{2p} \rightarrow S_p \Vert \Vert z \Vert _{p}. \end{aligned}$$

Hence we have obtained

$$\begin{aligned} \Vert M_{f^{[2]}}: S_{2p} \times S_{2p} \rightarrow S_{p} \Vert \gtrsim p^*. \end{aligned}$$

$$\square $$

### Remark 8.5

We argue that our result of Theorem 8.1 is fundamentally better than the methods employed in [11]. In principle, the method of proof in [11] can be adjusted to yield that $\Vert M_{f^{[2]}}: S_{2p} \times S_{2p} \rightarrow S_{p} \Vert \gtrsim pp^*$ for the same function *f* as in Theorems 8.1 and 8.4. Indeed, the idea of [11] is to first prove the reduction inequality

$$\begin{aligned} \Vert M_{f^{[2]}}: S_{2p} \times S_{2p} \rightarrow S_{p} \Vert \ge \sup _{\lambda _1 \in {\mathbb {R}}} \Vert M_{f^{[2]}(\, \cdot \,, \lambda _1, \, \cdot \,)}: S_{p} \rightarrow S_{p} \Vert . \end{aligned}$$

The right hand side has order $O(p p^*),$ which can be seen from Theorem 4.1 for instance. So the reduction of [11] is not efficient enough to capture the optimal constants.

## Data Availability

Data sharing is not applicable to this article as no datasets were generated or analysed during the current study.

## Appendix A: Proof of Theorem 6.5 following [15]

### A.1 Dyadic definitions and notations

We first give a brief overview over the dyadic notions used in the proof of Theorem 6.5. Unless noted otherwise, all definitions are from [15, Section 2.2]. While the concepts introduced in this section are well-defined on ${\mathbb {R}}^d,$ we restrict our discussion to $d=1,$ as it simplifies the notation and is in fact the only relevant case to our special case of Theorem 6.5.

See e.g. [15, Section 2.2] for the definitions on ${\mathbb {R}}^d$ for $d>1.$

**Dyadic grids.** The *standard dyadic grid* on ${\mathbb {R}}$ is defined as

$$\begin{aligned} {\mathcal {D}}_0\;:=\; \{2^{-k}([0,1)+m)\mid k,m\in {\mathbb {Z}}\}. \end{aligned}$$

Let $\Omega =\{0,1\}^{{\mathbb {Z}}}$ and equip $\Omega$ with a probability measure such that its coordinates are independent and uniformly distributed on $\{0,1\}.$ The *random dyadic grid* on ${\mathbb {R}}$ associated with ${\omega =(\omega _k)_{k\in {\mathbb {Z}}}\in \Omega }$ is defined as

$$\begin{aligned} {\mathcal {D}}_{\omega }&:= \{Q+\omega \mid Q\in {\mathcal {D}}_0\},\\ Q+\omega&:= Q+\sum _{\begin{array}{c} k\in {\mathbb {Z}}\\ 2^{-k}<|Q| \end{array}}2^{-k}\omega _k, \end{aligned}$$

where |*Q*| denotes the length of the cube *Q* in ${\mathbb {R}}.$ By a *dyadic grid* ${\mathcal {D}}$ we refer to ${\mathcal {D}}={\mathcal {D}}_{\omega }$ for some $\omega \in \Omega .$ For $Q\in {\mathcal {D}},$ ${\mathcal {D}}$ dyadic grid, define $Q^{(k)}$ as the cube $R\in {\mathcal {D}}$ such that $Q\subset R$ and $2^k|Q|=|R|.$ Further set ${\textrm{ch}}_{{\mathcal {D}}}(Q):= \{Q'\in {\mathcal {D}}\mid Q'\subsetneq Q \text { and there exists no } Q''\in {\mathcal {D}}\text { such that } Q' \subsetneq Q'' \subsetneq Q\}.$ We refer to this set as the *children* of *Q* in ${\mathcal {D}}.$ The index denoting the dyadic grid may be omitted.

**Haar functions.** Let ${\mathcal {D}}$ be a dyadic grid on ${\mathbb {R}}$ and let $Q\in {\mathcal {D}}.$ Let $Q_{\textrm{left}}$ (resp. $Q_{\textrm{right}}$) denote the left (resp. right) half of *Q*. For $\eta \in \{0,1\},$ we define the *Haar function*

$$\begin{aligned} h^{\eta }_Q\;:=\; {\left\{ \begin{array}{ll} |Q|^{-1/2}1_{Q}, &  \eta =0,\\ |Q|^{-1/2}(1_{Q_{\textrm{left}}}-1_{Q_{\textrm{right}}}), &  \eta =1. \end{array}\right. } \end{aligned}$$

To simplify the notation, we set $h_Q\;:=\;h_Q^{1}.$ Note that $\int _{{\mathbb {R}}}h_Q(x)dx=0,$ hence we refer to $h_Q$ as a *cancellative Haar function*. Furthermore, note that for any $Q_0\in {\mathcal {D}},$ an orthonormal basis of $L^2(Q_0)$ is given by the family $\{h^0_{Q_0}\}\cup \{h_Q\mid Q\subseteq Q_0\text { dyadic cube}\}.$

From the Haar functions we construct the *dyadic martingale difference* of a locally integrable function *f* as $(D_Q f)_Q,$ where

$$\begin{aligned} D_Qf= \langle f\rangle _{Q_{\textrm{left}}}1_{Q_{\textrm{left}}} + \langle f\rangle _{Q_{\textrm{right}}}1_{Q_{\textrm{right}}} - \langle f\rangle _{Q}1_{Q} = \langle f, h_Q\rangle h_Q, \end{aligned}$$

$\langle f \rangle _Q$ denotes the average of *f* over a region *Q*,  and $\langle f, g\rangle := \int _{{\mathbb {R}}} f(x)g(x)dx.$ Further define

$$\begin{aligned} \Delta _Q^lf:=\sum _{\begin{array}{c} R\in {\mathcal {D}}\\ R^{(l)}=Q \end{array}}D_Rf=\sum _{\begin{array}{c} R\in {\mathcal {D}}\\ R^{(l)}=Q \end{array}}\sum _{R'\in {\textrm{ch}}_{{\mathcal {D}}}(R)}(\langle f\rangle _{R'}-\langle f\rangle _{R})1_{R'}. \end{aligned}$$

**Shifts, paraproducts, and representation of Calderón–Zygmund Operators.** The proof of Theorem 6.5 heavily relies on a dyadic representation theorem for Calderón–Zygmund operators, see [30]. For the convenience of the reader, we repeat the relevant definitions here; see [15] for the general *n*-linear case.

Let *X* be a Banach space and ${\mathcal {D}}$ a dyadic grid on ${\mathbb {R}}.$ A *bilinear dyadic shift* $S_{{\mathcal {D}}}^k$ of complexity $k=(k_1,k_2,k_3)\in {\mathbb {N}}^{3}_0$ is defined on ${f,g\in L^{\infty }_c({\mathbb {R}},X)}$ as

A.1 $$\begin{aligned}&S_{{\mathcal {D}}}^{k}(f,g):=\sum _{Q\in {\mathcal {D}}}A_Q^{k}(f,g), \end{aligned}$$

A.2 $$\begin{aligned}&A_Q^k(f,g):=\sum _{\begin{array}{c} I_1,I_2,I_{3}\subseteq Q \\ |I_j|=2^{-k_j}|Q| \end{array}} \alpha _{I_1,I_2,I_{3},Q}\langle f,\tilde{h}_{I_1}\rangle \langle g,\tilde{h}_{I_2}\rangle \tilde{h}_{I_{3}}, \end{aligned}$$

where exactly one of $\tilde{h}_{I_{1}},\tilde{h}_{I_{2}},\tilde{h}_{I_{3}}$ is a non-cancellative Haar function and the other two are cancellative Haar functions. The index corresponding to the cancellative Haar function is denoted by $j_0.$ Furthermore, the coefficients $\alpha _{I_1,I_2,I_3,Q} \in {\mathbb {C}}$ must satisfy

A.3 $$\begin{aligned} |\alpha _{I_1,I_2,I_3,Q}|\le \frac{1}{|Q|^{2}} \prod _{j=1}^{3}|I_j|^{1/2}. \end{aligned}$$

A *bilinear paraproduct* is defined on $f,g\in L^{\infty }_c({\mathbb {R}})$ as

$$\begin{aligned} \pi _{{\mathcal {D}}}(f,g):= \sum _{Q\in {\mathcal {D}}}a_Q\langle f,\tilde{h}_{1,Q}\rangle \langle g,\tilde{h}_{2,Q}\rangle \tilde{h}_{3,Q}, \end{aligned}$$

where $(\tilde{h}_{1,Q},\tilde{h}_{2,Q},\tilde{h}_{3,Q})$ are such that there is exactly one $j_0\in \{1,2,3\}$ such that for all $Q\in {\mathcal {D}}$ we have $\tilde{h}_{j_0,Q}=h_{Q}$ and $\tilde{h}_{j,Q}=1_{Q}/|Q|$ for all $j\ne j_0.$ The scalar sequence $(a_Q)_{Q\in {\mathcal {D}}}$ is such that

$$\begin{aligned} \sup _{Q_0\in {\mathcal {D}}}\left( \frac{1}{|Q_0|}\sum _{\begin{array}{c} Q\in {\mathcal {D}}\\ Q\subset Q_0 \end{array}}|a_Q|^2\right) ^{1/2}\le 1. \end{aligned}$$

Note that we will usually suppress the dyadic grid from the notation and refer to dyadic shifts and paraproducts as $S^k$ and $\pi ,$ respectively.

Let *T* be a bilinear Calderón–Zygmund operator and $f,g,h\in L^{\infty }_c({\mathbb {R}}).$ Then

A.4 $$\begin{aligned} \langle T(f,g),h\rangle = C_T {\mathbb {E}}_{\omega }\sum _{k\in {\mathbb {N}}_0^{3}}\sum _u2^{-\max _i k_i/2}\langle U_{{\mathcal {D}}_{\omega },u}^k(f,g),h\rangle , \end{aligned}$$

where $C_T$ is a constant depending only on *T*,  the sum over *u* is finite, and ${\mathcal {D}}_{\omega }$ is a random dyadic grid. For $\max _j k_j>0,$ $U_{{\mathcal {D}}_{\omega },u}^k$ denotes a bilinear dyadic shift of complexity *k*,  whereas for ${\max _j k_j=0},$ $U_{{\mathcal {D}}_{\omega },u}^k$ denotes either a bilinear dyadic shift of complexity 0 or a bilinear paraproduct. Note that by Equation (4.4) in [30], the paraproducts in this representation are constructed from a scalar sequence

$$\begin{aligned} a_Q=C_{T}\langle T(1,1),h_Q\rangle , \end{aligned}$$

hence $T(1,1)=0$ implies that the paraproducts in the representation of *T* vanish. This applies in particular to the situation of Remark 6.4, where we have

A.5 $$\begin{aligned} \langle T(f,g),h\rangle = C_T {\mathbb {E}}_{\omega }\sum _{k\in {\mathbb {N}}_0^{3}}\sum _u2^{-\max _i k_i/2}\langle S_{{\mathcal {D}}_{\omega },u}^k(f,g),h\rangle . \end{aligned}$$

### A.2 Relevant inequalities

Before presenting the proof of Theorem 6.5, we first list the estimates that will be used, alongside the constants they introduce.

Following [15], it is sufficient to consider the following special case of the decoupling estimate [22, Theorem 6].

#### Theorem A.1

(Decoupling Inequality [22, Theorem 6]) Let $p\in (1,\infty ),$ let *X* be a UMD space with UMD constant $\beta _{p,X},$ and let ${\mathcal {D}}$ be a dyadic grid. Further define the following : 

- $${\mathcal {D}}_{j,k}:=\{Q\in {\mathcal {D}}\mid |Q|=2^{m(k+1)+j}\text { for some }m\in {\mathbb {Z}}\}$$ for $$j,k\in {\mathbb {Z}}$$ fixed, 
- the probability space ${\mathcal {V}}_Q:=(Q,\textrm{Leb}(Q),\lambda _Q),$ where $\textrm{Leb}(Q)$ denotes the Lebesgue measurable subsets of *Q* and $\lambda _Q$ the normalised restriction of the Lebesgue measure to *Q*, 
- the product probability space ${\mathcal {V}}:=\prod _{Q\in {\mathcal {D}}}{\mathcal {V}}_Q$ with measure $\nu$ and elements $y=(y_Q)_{Q\in {\mathcal {D}}}.$

Let $(\varepsilon _Q)_{Q\in {\mathcal {D}}}$ be a Rademacher sequence. Let $(f_Q)_{Q\in {\mathcal {D}}}$ be a sequence of functions ${\mathbb {R}} \rightarrow X$ such that for all $Q\in {\mathcal {D}},$ $f_Q$ is (1) supported on *Q*,  (2) constant on every $Q'\in {\textrm{ch}}_{{\mathcal {D}}}(Q),$ and (3) $\langle f_Q\rangle _Q = 0$ holds. Then

A.6 $$\begin{aligned}  &   \frac{1}{\beta _{p,X}^p}{\mathbb {E}}\int _{{\mathbb {R}}} \int _{{\mathcal {V}}}\Vert \sum _{Q\in {\mathcal {D}}_{j,k}}\varepsilon _Q1_Q(x) f_Q(y_Q)\Vert _{X}^pd\nu (y)dx \nonumber \\  &   \quad \le \int _{{\mathbb {R}}} \Vert \sum _{Q\in {\mathcal {D}}_{j,k}}f_Q(x)\Vert _{X}^pdx \le \beta _{p,X}^p {\mathbb {E}}\int _{{\mathbb {R}}} \int _{{\mathcal {V}}}\Vert \sum _{Q\in {\mathcal {D}}_{j,k}}\varepsilon _Q1_Q(x) f_Q(y_Q)\Vert _{X}^pd\nu (y)dx.\nonumber \\ \end{aligned}$$

This inequality also holds when replacing ${\mathcal {D}}_{j,k}$ with ${\mathcal {D}}.$

#### Theorem A.2

(Kahane–Khintchine inequality, [23, Theorem 3.2.23]) Let $(\varepsilon _n)_n$ be a Rademacher sequence on a probability space $\Omega ,$ and let *X* be a Banach space. For $p,q\in (0,\infty )$ there exists $\kappa _{p,q}<\infty$ such that for all $N\in {\mathbb {N}}$ and $x_1,\dotsc ,x_N\in X$ we have

$$\begin{aligned} \Vert \sum _{n=1}^N\varepsilon _nx_n\Vert _{L^p(\Omega ,X)}\le \kappa _{p,q}\Vert \sum _{n=1}^N\varepsilon _nx_n\Vert _{L^q(\Omega ,X)}. \end{aligned}$$

#### Remark A.3

Relevant in this section is the case $p=2,$ $q>1.$ Following the proof of Theorem A.2 in [23], the constant $\kappa _{p,q}$ is the same as in [23, Theorem 3.2.17] for $p,q\ge 1,$ namely $\kappa _{p,q}=2^{1+1/q}e\left( 1+2\frac{p}{q}\right) .$ In particular, we thus have $\kappa _{2,q}\le 12(1+4/q)\le 60$ for all $q\ge 1.$

The following theorem has been specialised to our dyadic setting. Stein’s inequality is originally due to Bourgain, and for the explicit constant we refer to the proof in [17] which is also contained in the monograph [23].

#### Theorem A.4

(Stein’s inequality, Eqn. (2.3) of [15], Theorem 4.2.23 of [23], or Lemma 34 of [17]) Let *X* be a UMD space with UMD constant $\beta _{p,X}$ and let ${\mathcal {D}}$ be a dyadic grid. Let $(f_Q)_{ Q\in {\mathcal {D}}}$ be a sequence in $L^1_{\textrm{loc}}(X)$ such that ${\textrm{supp}}\; f_Q\subseteq Q,$ $Q\in {\mathcal {D}},$ and such that only finitely many of them are nonzero, and let $p\in (1,\infty ).$ Then

$$\begin{aligned} {\mathbb {E}}\Vert \sum _{Q\in {\mathcal {D}}}\varepsilon _Q\langle f_Q\rangle _Q1_Q\Vert _{L^p({\mathbb {R}},X)}\le \beta _{p,X} {\mathbb {E}}\Vert \sum _{Q\in {\mathcal {D}}}\varepsilon _Qf_Q\Vert _{L^p({\mathbb {R}},X)}. \end{aligned}$$

#### Theorem A.5

(Kahane contraction principle, [23, Proposition 3.2.10]) Let $(\varepsilon _n)_n$ be a Rademacher sequence on a probability space $\Omega ,$ $(a_n)_n$ a finite scalar sequence,  and $(x_n)_n$ a finite sequence in a Banach space *X*. Let $1\le p\le \infty .$ Then

$$\begin{aligned} \Vert \sum _{n=1}^Na_n\varepsilon _nx_n\Vert _{L^p(\Omega ;X)} \le \max _{n}|a_n|\Vert \sum _{n=1}^N\varepsilon _nx_n\Vert _{L^p(\Omega ;X)}. \end{aligned}$$

### A.3 Proof of Theorem 6.5

We repeat the proof of Theorem 3.17 in [15], specialised to the bilinear case for $d=1$ and $T(1,1)=0$ (in the sense of Remark 6.4). By the representation theorem introduced in Appendix A.1, the proof reduces to the following theorem from [15, Section 4].

#### Theorem A.6

Let $p,p_1,p_2\in (1,\infty )$ such that $1/p_1+1/p_2=1/p.$ Set $p_3:=p^*.$ Let $S^k$ be a bilinear dyadic shift of complexity $k=(k_1,k_2,k_3)\in {\mathbb {N}}^{3}_0$ and let $f_j\in L^{\infty }_c({\mathbb {R}},S_{p_j}),$ $j=1,2,3.$ Define the associated trilinear form

$$\begin{aligned} \Lambda _{S^k}(f_1,f_2,f_3)=\sum _{Q\in {\mathcal {D}}}\sum _{\begin{array}{c} I_1,I_2,I_{3}\subseteq Q \\ |I_j|=2^{-k_j}|Q| \end{array}} \alpha _{I_1,I_2,I_{3},Q}\tau \left( \langle f_1,\tilde{h}_{I_1}\rangle \langle f_2,\tilde{h}_{I_2}\rangle \langle f_3, \tilde{h}_{I_{3}}\rangle \right) , \end{aligned}$$

where $\tau$ denotes the trace. It then holds that

A.7 $$\begin{aligned} |\Lambda _{S^k}(f_1,f_2,f_3)| \lesssim C(p,p_1,p_2)\prod _{j=1}^3\Vert f_j\Vert _{L^{p_j}({\mathbb {R}},S_{p_j})}. \end{aligned}$$

#### Proof

The trilinear form is first rewritten as

A.8 $$\begin{aligned} \Lambda _{S^k}(f_1,f_2,f_3)&= \sum _{i=0}^{\kappa } \Lambda _{S_i^k}(f_1,f_2,f_3), \end{aligned}$$

A.9 $$\begin{aligned} \Lambda _{S_i^k}(f_1,f_2,f_3)&=\sum _{K\in {\mathcal {D}}_{i,\kappa }}\sum _{\begin{array}{c} L_1,L_2,L_3\in {\mathcal {D}}\\ L_j^{(l_j)}=K \end{array}}b_{L_1,L_2,L_3,K}\tau \left( \prod _{j=1}^3\langle f_j,h'_{L_j}\rangle \right) , \end{aligned}$$

A.10 $$\begin{aligned} b_{L_1,L_2,L_3,K}&= \sum _{\begin{array}{c} Q_1,Q_2,Q_3\in {\mathcal {D}}\\ Q_j^{(k_j-l_j)}=L_j \end{array}}a_{Q_1,Q_2,Q_3,K}\prod _{j=1}^3\frac{|Q_j|^{1/2}}{|L_j|^{1/2}}, \end{aligned}$$

where $0\le l_j\le k_j$ and $\kappa =\max k_j.$ This is a new shift operator with $h'_{L_j}\in \{h^0_{L_j},h_{L_j}\}$ such that there may be more than two indices *j* such that their associated Haar functions are cancellative, whereas in (A.2), the Haar functions are cancellative for exactly two indices. Furthermore, the construction is such that if $h'_{L_j}$ is not cancellative, then $l_j=0.$ For details on how to construct this new shift, see [15].

The proof now proceeds as follows. First, boundedness is shown in the case where all Haar functions $h'_{L_j}$ are cancellative. In the second case, where not all Haar functions are cancellative, the fact $h'_{L_j}=h^0_{L_j} \Rightarrow l_j=0$ allows us to reduce the trilinear form (A.9) to a bilinear form with only cancellative Haar functions. For this new bilinear form, boundedness follows by the same proof method as in the first case.

**Case 1.** Let $0\le i\le \kappa$ be such that all associated Haar functions in (A.9) are cancellative. Note that for $L_3^{(l_3)}\in {\mathcal {D}}_{i,\kappa },$ orthogonality of the Haar functions yields

$$\begin{aligned} \sum _{K\in {\mathcal {D}}_{i,\kappa }}\Delta _K^{l_3}h_{L_3} = \sum _{K\in {\mathcal {D}}_{i,\kappa }} \sum _{\begin{array}{c} L\in {\mathcal {D}}\\ L^{(l_3)}=K \end{array}} D_L h_{L_3} = \sum _{K\in {\mathcal {D}}_{i,\kappa }} \sum _{\begin{array}{c} L\in {\mathcal {D}}\\ L^{(l_3)}=K \end{array}} \langle h_{L_3}, h_L\rangle h_L =h_{L_3}. \end{aligned}$$

Using the decoupling inequality from Theorem A.1, we thus have

$$\begin{aligned}  &   \Vert S^k_i(f_1,f_2)\Vert _{L^p({\mathbb {R}},S_p)} \\  &   \quad = \Vert \sum _{K\in {\mathcal {D}}_{i,\kappa }}\sum _{\begin{array}{c} L_1,L_2,L_3\in {\mathcal {D}}\\ L_j^{(l_j)}=K \end{array}}b_{L_1,L_2,L_3,K}\langle f_1,h_{L_1}\rangle \langle f_2,h_{L_2}\rangle h_{L_3}\Vert _{L^p({\mathbb {R}},S_p)} \\  &   \quad \le \beta _{p,S_p}({\mathbb {E}}\int _{{\mathbb {R}}}\int _{{\mathcal {V}}}\Vert \sum _{K\in {\mathcal {D}}_{i,\kappa }}\varepsilon _K1_K(x)\sum _{\begin{array}{c} L_1,L_2,L_3\in {\mathcal {D}}\\ L_j^{(l_j)}=K \end{array}}b_{L_1,L_2,L_3,K}\\  &   \prod _{j=1}^2\langle f_j,h_{L_j}\rangle h_{L_3}(y_K)\Vert ^p_{S_p}d\nu (y)dx)^{1/p}. \end{aligned}$$

We can rewrite the inner sum in the integral by using $\langle f_j,h_{L_j}\rangle =\langle \Delta ^{l_j}_Kf_j,h_{L_j}\rangle .$ Indeed,

$$\begin{aligned} \langle \Delta ^{l_j}_Kf_j,h_{L_j}\rangle = \sum _{\begin{array}{c} L\in {\mathcal {D}}\\ L^{(l_j)}=K \end{array}} \langle D_Lf_j, h_{L_j}\rangle = \sum _{\begin{array}{c} L\in {\mathcal {D}}\\ L^{(l_j)}=K \end{array}} \langle f_j, h_{L}\rangle \langle h_L, h_{L_j}\rangle = \langle f_j, h_{L_j}\rangle . \end{aligned}$$

Hence we can write

$$\begin{aligned}&\sum _{\begin{array}{c} L_1,L_2,L_3\in {\mathcal {D}}\\ L_j^{(l_j)}=K \end{array}}b_{L_1,L_2,L_3,K}\prod _{j=1}^2\langle f_j,h_{L_j}\rangle h_{L_3}(y_K) \\&\qquad =\sum _{\begin{array}{c} L_1,L_2,L_3\in {\mathcal {D}}\\ L_j^{(l_j)}=K \end{array}}b_{L_1,L_2,L_3,K}\prod _{j=1}^2\langle \Delta ^{l_j}_Kf_j,h_{L_j}\rangle h_{L_3}(y_K) \\&\qquad = \int _{K^2}\sum _{\begin{array}{c} L_1,L_2,L_3\in {\mathcal {D}}\\ L_j^{(l_j)}=K \end{array}}b_{L_1,L_2,L_3,K}\prod _{j=1}^2\Delta ^{l_j}_Kf_j(z_j)h_{L_j}(z_j) h_{L_3}(y_K)dz. \end{aligned}$$

By setting

$$\begin{aligned} b_K(y_K,z)=|K|^2\sum _{\begin{array}{c} L_1,L_2,L_3\in {\mathcal {D}}\\ L_j^{(l_j)}=K \end{array}}b_{L_1,L_2,L_3,K}\prod _{j=1}^2h_{L_j}(z_{j}) h_{L_3}(y_K), \end{aligned}$$

we have

$$\begin{aligned}  &   \int _{K^2}\sum _{\begin{array}{c} L_1,L_2,L_3\in {\mathcal {D}}\\ L_j^{(l_j)}=K \end{array}}b_{L_1,L_2,L_3,K}\prod _{j=1}^2\Delta ^{l_j}_Kf_j(z_j)h_{L_j}(z_j) h_{L_3}(y_K)dz\\  &   \quad =\frac{1}{|K|^2}\int _{K^2}b_K(y_K,z)\prod _{j=1}^2\Delta ^{l_j}_Kf_j(z_j)dz=\int _{{\mathcal {V}}^2}b_K(y_K,z_K)\prod _{j=1}^2\Delta ^{l_j}_Kf_j(z_{j,K})d\nu (z), \end{aligned}$$

where ${\mathcal {V}}$ and $\nu$ are as defined in Theorem A.1. We can use the triangle inequality and as ${\mathcal {V}}^2$ is a probability space then apply Jensen’s inequality to show

$$\begin{aligned}&\Vert S^k_i(f_1,f_2)\Vert _{L^p({\mathbb {R}},S_p)} \\&\quad \le \beta _{p,S_p}\left( {\mathbb {E}}\int _{{\mathbb {R}}}\int _{{\mathcal {V}}}\Vert \int _{{\mathcal {V}}^2}\sum _{K\in {\mathcal {D}}_{i,\kappa }}\varepsilon _K1_K(x)b_K(y_K,z_K)\prod _{j=1}^2\Delta ^{l_j}_Kf_j(z_{j,K})d\nu (z)\Vert ^p_{S_p}d\nu (y)dx\right) ^{1/p} \\&\quad \le \beta _{p,S_p}\left( {\mathbb {E}}\int _{{\mathbb {R}}}\int _{{\mathcal {V}}}\left( \int _{{\mathcal {V}}^2}\Vert \sum _{K\in {\mathcal {D}}_{i,\kappa }}\varepsilon _K1_K(x)b_K(y_K,z_K)\prod _{j=1}^2\Delta ^{l_j}_Kf_j(z_{j,K})\Vert _{S_p}d\nu (z)\right) ^pd\nu (y)dx\right) ^{1/p} \\&\quad \le \beta _{p,S_p}\left( {\mathbb {E}}\int _{{\mathbb {R}}}\int _{{\mathcal {V}}}\int _{{\mathcal {V}}^2}\Vert \sum _{K\in {\mathcal {D}}_{i,\kappa }}\varepsilon _K1_K(x)b_K(y_K,z_K)\prod _{j=1}^2\Delta ^{l_j}_Kf_j(z_{j,K})\Vert ^p_{S_p}d\nu (z)d\nu (y)dx\right) ^{1/p}. \end{aligned}$$

Note that by construction, $|b_K(y_K,z_K)|\le 1.$ Indeed, by unfolding definitions and applying estimate (A.3) we have

$$\begin{aligned}&|b_K(y_K,z_K)|\\&\quad \le |K|^2\sum _{\begin{array}{c} L_1,L_2,L_3\in {\mathcal {D}}\\ L_j^{(l_j)}=K \end{array}}|b_{L_1,L_2,L_3,K}|\prod _{j=1}^2|h_{L_j}(z_{j,K})| |h_{L_3}(y_K)| \\&\quad \!\!\le \!\! |K|^2\sum _{\begin{array}{c} L_1,L_2,L_3\!\in \!{\mathcal {D}}\\ L_j^{(l_j)}\!=\!K \end{array}}\sum _{\begin{array}{c} Q_1,Q_2,Q_3\!\in \!{\mathcal {D}}\\ Q_j^{(k_j-l_j)}\!=\!L_j \end{array}}|a_{Q_1,Q_2,Q_3,K}|\prod _{j\!=\!1}^3\frac{|Q_j|^{1/2}}{|L_j|^{1/2}}\frac{1_{L_1}(z_{1,K})}{|L_1|^{1/2}}\frac{1_{L_2}(z_{2,K})}{|L_2|^{1/2}} \frac{1_{L_3}(y_{K})}{|L_3|^{1/2}} \\&\quad \le \sum _{\begin{array}{c} L_1,L_2,L_3\in {\mathcal {D}}\\ L_j^{(l_j)}=K \end{array}}\sum _{\begin{array}{c} Q_1,Q_2,Q_3\in {\mathcal {D}}\\ Q_j^{(k_j-l_j)}=L_j \end{array}}\prod _{l=1}^3|Q_l|^{1/2}\prod _{j=1}^3\frac{|Q_j|^{1/2}}{|L_j|}1_{L_1}(z_{1,K})1_{L_2}(z_{2,K})1_{L_3}(y_K). \end{aligned}$$

Since the size of $|Q_j|$ relative to $|L_j|$ is fixed, we can rewrite this expression as

$$\begin{aligned}  &   \sum _{\begin{array}{c} L_1,L_2,L_3\in {\mathcal {D}}\\ L_j^{(l_j)}=K \end{array}}\sum _{\begin{array}{c} Q_1,Q_2,Q_3\in {\mathcal {D}}\\ Q_j^{(k_j-l_j)}=L_j \end{array}}\prod _{l=1}^3|Q_l|^{1/2}\prod _{j=1}^3\frac{|Q_j|^{1/2}}{|L_j|}1_{L_1}(z_{1,K})1_{L_2}(z_{2,K})1_{L_3}(y_K).\\  &   \quad =\sum _{\begin{array}{c} L_1,L_2,L_3\in {\mathcal {D}}\\ L_j^{(l_j)}=K \end{array}}\sum _{\begin{array}{c} Q_1,Q_2,Q_3\in {\mathcal {D}}\\ Q_j^{(k_j-l_j)}=L_j \end{array}}\prod _{j=1}^32^{l_j-k_j}1_{L_1}(z_{1,K})1_{L_2}(z_{2,K})1_{L_3}(y_K). \end{aligned}$$

Finally, we use

$$\begin{aligned} \sum _{\begin{array}{c} Q_1,Q_2,Q_3\in {\mathcal {D}}\\ Q_j^{(k_j-l_j)}=L_j \end{array}} 1= \prod _{j=1}^32^{k_j-l_j} \end{aligned}$$

and the disjointness of the children of *K* to conclude

$$\begin{aligned}&\sum _{\begin{array}{c} L_1,L_2,L_3\in {\mathcal {D}}\\ L_j^{(l_j)}=K \end{array}}\sum _{\begin{array}{c} Q_1,Q_2,Q_3\in {\mathcal {D}}\\ Q_j^{(k_j-l_j)}=L_j \end{array}}\prod _{j=1}^32^{l_j-k_j}1_{L_1}(z_{1,K})1_{L_2}(z_{2,K})1_{L_3}(y_K) \\&\quad =\sum _{\begin{array}{c} L_1,L_2,L_3\in {\mathcal {D}}\\ L_j^{(l_j)}=K \end{array}}\prod _{j=1}^32^{k_j-l_j}2^{l_j-k_j}1_{L_1}(z_{1,K})1_{L_2}(z_{2,K})1_{L_3}(y_K)\\&\quad =\sum _{\begin{array}{c} L_1,L_2,L_3\in {\mathcal {D}}\\ L_j^{(l_j)}=K \end{array}}1_{L_1}(z_{1,K})1_{L_2}(z_{2,K})1_{L_3}(y_K)\\&\quad =1_{K}(z_{1,K})1_{K}(z_{2,K})1_{K}(y_K)\\&\quad \le 1. \end{aligned}$$

Letting $\Vert (x_k)_{k=1}^K\Vert _{\textrm{Rad}(S_{p})}:=\left( {\mathbb {E}}\Vert \sum _{k=1}^K\varepsilon _kx_k\Vert _{S_{p}}^2\right) ^{1/2},$ we can now finish the proof of this case as follows. From the previous estimates and [15, Lemma 4.1], it follows that

$$\begin{aligned}&\Vert S^k_i(f_1,f_2)\Vert _{L^p({\mathbb {R}},S_p)} \\&\quad {\displaystyle \le \beta _{p,S_p}\left( {\mathbb {E}}\int _{{\mathbb {R}}}\int _{{\mathcal {V}}}\int _{{\mathcal {V}}^2}\Vert \sum _{K\in {\mathcal {D}}_{i,\kappa }}\varepsilon _K1_K(x)b_K(y_K,z_K)\prod _{j=1}^2\Delta ^{l_j}_Kf_j(z_{j,K})\Vert ^p_{S_p}d\nu (z)d\nu (y)dx\right) ^{1/p}} \\&\quad {\displaystyle \le \beta _{p,S_p}\left( \int _{{\mathbb {R}}}\int _{{\mathcal {V}}}\int _{{\mathcal {V}}^2}\prod _{j=1}^2\Vert (1_K(x)\Delta ^{l_j}_Kf_j(z_{j,K}))_{K\in {\mathcal {D}}_{i,\kappa }}\Vert ^p_{\textrm{Rad}(S_{p_j})}d\nu (z)d\nu (y)dx\right) ^{1/p}}. \end{aligned}$$

Using that ${\mathcal {V}}$ is a probability space and applying Hölder’s inequality yields

$$\begin{aligned}&\beta _{p,S_p}\left( \int _{{\mathbb {R}}}\int _{{\mathcal {V}}}\int _{{\mathcal {V}}^2}\prod _{j=1}^2\Vert (1_K(x)\Delta ^{l_j}_Kf_j(z_{j,K}))_{K\in {\mathcal {D}}_{i,\kappa }}\Vert ^p_{\textrm{Rad}(S_{p_j})}d\nu (z)d\nu (y)dx\right) ^{1/p} \\&\quad {\displaystyle = \beta _{p,S_p}\left( \int _{{\mathbb {R}}}\int _{{\mathcal {V}}^2}\prod _{j=1}^2\Vert (1_K(x)\Delta ^{l_j}_Kf_j(z_{j,K}))_{K\in {\mathcal {D}}_{i,\kappa }}\Vert ^p_{\textrm{Rad}(S_{p_j})}d\nu (z)dx\right) ^{1/p}} \\&\quad {\displaystyle \le \beta _{p,S_p}\prod _{j=1}^2\left( \int _{{\mathbb {R}}}\int _{{\mathcal {V}}^2}\Vert (1_K(x)\Delta ^{l_j}_Kf_j(z_{j,K}))_{K\in {\mathcal {D}}_{i,\kappa }}\Vert ^{p_j}_{\textrm{Rad}(S_{p_j})}d\nu (z)dx\right) ^{1/p_j}}. \end{aligned}$$

By unfolding the definition of $\Vert \cdot \Vert _{\textrm{Rad}},$ we can apply the Kahane–Khintchine equality to obtain

$$\begin{aligned}&\beta _{p,S_p}\prod _{j=1}^2\left( \int _{{\mathbb {R}}}\int _{{\mathcal {V}}^2}\Vert (1_K(x)\Delta ^{l_j}_Kf_j(z_{j,K}))_{K\in {\mathcal {D}}_{i,\kappa }}\Vert ^{p_j}_{\textrm{Rad}(S_{p_j})}d\nu (z)dx\right) ^{1/p_j} \\&\quad {\displaystyle = \beta _{p,S_p}\prod _{j=1}^2\left( \int _{{\mathbb {R}}}\int _{{\mathcal {V}}^2}({\mathbb {E}}\Vert \sum _{K\in {\mathcal {D}}_{i,\kappa }}\varepsilon _K1_K(x)\Delta ^{l_j}_Kf_j(z_{j,K})\Vert _{S_{p_j}}^2)^{p_j/2}d\nu (z)dx\right) ^{1/p_j}} \\&\quad {\displaystyle \le \beta _{p,S_p}\prod _{j=1}^2\kappa _{2,p_j}\left( \int _{{\mathbb {R}}}\int _{{\mathcal {V}}^2}{\mathbb {E}}\Vert \sum _{K\in {\mathcal {D}}_{i,\kappa }}\varepsilon _K1_K(x)\Delta ^{l_j}_Kf_j(z_{j,K})\Vert _{S_{p_j}}^{p_j}d\nu (z)dx\right) ^{1/p_j}}. \end{aligned}$$

Finally, Fubini’s theorem and the decoupling estimate yield

$$\begin{aligned}&\beta _{p,S_p}\prod _{j=1}^2\kappa _{2,p_j}\left( \int _{{\mathbb {R}}}\int _{{\mathcal {V}}^2}{\mathbb {E}}\Vert \sum _{K\in {\mathcal {D}}_{i,\kappa }}\varepsilon _K1_K(x)\Delta ^{l_j}_Kf_j(z_{j,K})\Vert _{S_{p_j}}^{p_j}d\nu (z)dx\right) ^{1/p_j} \\&\quad {\displaystyle =\beta _{p,S_p}\prod _{j=1}^2\kappa _{2,p_j}\left( {\mathbb {E}}\int _{{\mathbb {R}}}\int _{{\mathcal {V}}^2}\Vert \sum _{K\in {\mathcal {D}}_{i,\kappa }}\varepsilon _K1_K(x)\Delta ^{l_j}_Kf_j(z_{j,K})\Vert _{S_{p_j}}^{p_j}d\nu (z)dx\right) ^{1/p_j}} \\&\quad {\displaystyle \le \beta _{p,S_p}\prod _{j=1}^2\kappa _{2,p_j}\beta _{p_j,S_{p_j}}\Vert f_j\Vert _{L^{p_j}({\mathbb {R}},S_{p_j})}}, \end{aligned}$$

concluding the proof of Case 1. Altogether, this case yields the estimate

$$\begin{aligned} \Vert S^k_i(f_1,f_2)\Vert _{L^p({\mathbb {R}},S_p)} \lesssim \beta _{p,S_p}\prod _{j=1}^2\kappa _{2,p_j}\beta _{p_j,S_{p_j}}. \end{aligned}$$

**Case 2.** Let $0\le i\le \kappa$ be such that one Haar function in (A.9) is not cancellative. We assume that $h'_{L_2}=h^0_{L_2}$ and $h'_{L_j}=h_{L_j},$ $j=1,3;$ the estimates for the other cases follow in the same manner. Note that (A.9) has been constructed such that this implies $l_2=0,$ hence $L_2=K;$ see [15] for details. We use the decoupling estimate (Theorem A.1) to estimate

$$\begin{aligned} \Vert S^k_i(f_1,f_2)\Vert _{L^p({\mathbb {R}},S_p)}&= \Vert \sum _{K\in {\mathcal {D}}_{i,\kappa }}\sum _{\begin{array}{c} L_1,L_3\in {\mathcal {D}}\\ L_j^{(l_j)}=K \end{array}}b_{L_1,L_3,K}\langle f_1,h_{L_1}\rangle |K|^{1/2}\langle f_2\rangle _Kh_{L_3}\Vert _{L^p({\mathbb {R}},S_p)} \\&\le \beta _{p,S_p}\left( {\mathbb {E}}\int _{{\mathbb {R}}}\int _{{\mathcal {V}}}\Vert \sum _{K\in {\mathcal {D}}_{i,\kappa }}\varepsilon _K1_K(x)\langle \varphi _{K,y}\rangle _K\Vert _{S_p}^pd\nu (y)dx\right) ^{1/p}, \end{aligned}$$

where the function $\varphi _{K,y}:{\mathbb {R}}\rightarrow S_p$ is defined as

$$\begin{aligned} \varphi _{K,y}(x)\;:=\;|K|^{1/2}\sum _{\begin{array}{c} L_1,L_3\in {\mathcal {D}}\\ L_j^{(l_j)}=K \end{array}}b_{L_1,L_3,K}\langle f_1,h_{L_1}\rangle f_2(x)h_{L_3}(y_K). \end{aligned}$$

We can now apply Stein’s inequality (Theorem A.4) with respect to $x\in {\mathbb {R}}$ to obtain

$$\begin{aligned}  &   \beta _{p,S_p}\left( {\mathbb {E}}\int _{{\mathbb {R}}}\int _{{\mathcal {V}}}\Vert \sum _{K\in {\mathcal {D}}_{i,\kappa }}\varepsilon _K1_K(x)\langle \varphi _{K,y}\rangle _K\Vert _{S_p}^pd\nu (y)dx\right) ^{1/p} \\  &   \quad \le \beta ^2_{p,S_p}\left( {\mathbb {E}}\int _{{\mathbb {R}}}\int _{{\mathcal {V}}}\Vert \sum _{K\in {\mathcal {D}}_{i,\kappa }}\varepsilon _K1_K(x)\varphi _{K,y}(x)\Vert _{S_p}^pd\nu (y)dx\right) ^{1/p}. \end{aligned}$$

By Hölder’s inequality we can further estimate

$$\begin{aligned}  &   \left( {\mathbb {E}}\int _{{\mathbb {R}}}\int _{{\mathcal {V}}}\Vert \sum _{K\in {\mathcal {D}}_{i,\kappa }}\varepsilon _K1_K(x)\varphi _{K,y}(x)\Vert _{S_p}^pd\nu (y)dx\right) ^{1/p}\\  &   \quad \le ({\mathbb {E}}\int _{{\mathbb {R}}}\int _{{\mathcal {V}}}\Vert \sum _{K\in {\mathcal {D}}_{i,\kappa }}\varepsilon _K1_K(x)|K|^{1/2}\sum _{\begin{array}{c} L_1,L_3\in {\mathcal {D}}\\ L_j^{(l_j)}=K \end{array}}b_{L_1,L_3,K}\langle f_1,h_{L_1}\rangle h_{L_3}(y_K)\Vert _{S_{p_1}}^p\\  &   \Vert f_2(x)\Vert _{S_{p_2}}^pd\nu (y)dx)^{1/p}\\  &   \quad \le ({\mathbb {E}}\int _{{\mathbb {R}}}\int _{{\mathcal {V}}}\Vert \sum _{K\in {\mathcal {D}}_{i,\kappa }}\varepsilon _K1_K(x)|K|^{1/2}\sum _{\begin{array}{c} L_1,L_3\in {\mathcal {D}}\\ L_j^{(l_j)}=K \end{array}}b_{L_1,L_3,K}\langle f_1,h_{L_1}\rangle h_{L_3}(y_K)\Vert _{S_{p_1}}^{p_1} \\  &   \times d\nu (y)dx)^{1/p_1}\Vert f_2\Vert _{L^{p_2}({\mathbb {R}},S_{p_2})}. \end{aligned}$$

We now proceed as in Case 1 to estimate the remaining term. We use

$$\begin{aligned} |K|^{1/2}\sum _{\begin{array}{c} L_1,L_3\in {\mathcal {D}}\\ L_j^{(l_j)}=K \end{array}}b_{L_1,L_3,K}\langle f_1,h_{L_1}\rangle h_{L_3}(y_K)&= \int _{{\mathcal {V}}}b_K(y_k,z_K)\Delta _K^{l_1}f_1(z_K)d\nu (z), \end{aligned}$$

where we define

$$\begin{aligned} b_K(y_k,z_K) =|K|^{3/2}\sum _{\begin{array}{c} L_1,L_3\in {\mathcal {D}}\\ L_j^{(l_j)}=K \end{array}}b_{L_1,L_3,K}h_{L_1}(z)h_{L_3}(y_K), \end{aligned}$$

and estimate the remaining integral as

$$\begin{aligned}&\Biggl ({\mathbb {E}}\int _{{\mathbb {R}}}\int _{{\mathcal {V}}}\Vert \sum _{K\in {\mathcal {D}}_{i,\kappa }}\varepsilon _K1_K(x)|K|^{1/2}\sum _{\begin{array}{c} L_1,L_3\in {\mathcal {D}}\\ L_j^{(l_j)}=K \end{array}}b_{L_1,L_3,K}\langle f_1,h_{L_1}\rangle h_{L_3}(y_K)\Vert _{S_{p_1}}^{p_1} d\nu (y)dx\Biggr )^{1/p_1}\\&\quad =\left( {\mathbb {E}}\int _{{\mathbb {R}}}\int _{{\mathcal {V}}}\Vert \sum _{K\in {\mathcal {D}}_{i,\kappa }}\varepsilon _K1_K(x)\int _{{\mathcal {V}}}b_K(y_k,z_K)\Delta _K^{l_1}f_1(z_K)d\nu (z)\Vert _{S_{p_1}}^{p_1} d\nu (y)dx\right) ^{1/p_1} \\&\quad \le \left( {\mathbb {E}}\int _{{\mathbb {R}}}\int _{{\mathcal {V}}}\int _{{\mathcal {V}}}\Vert \sum _{K\in {\mathcal {D}}_{i,\kappa }}\varepsilon _K1_K(x)b_K(y_k,z_K)\Delta _K^{l_1}f_1(z_K)\Vert _{S_{p_1}}^{p_1}d\nu (z) d\nu (y)dx\right) ^{1/p_1} \end{aligned}$$

Using Fubini’s theorem and the Kahane contraction principle (Theorem A.5) we further have

$$\begin{aligned}&\left( {\mathbb {E}}\int _{{\mathbb {R}}}\int _{{\mathcal {V}}}\int _{{\mathcal {V}}}\Vert \sum _{K\in {\mathcal {D}}_{i,\kappa }}\varepsilon _K1_K(x)b_K(y_k,z_K)\Delta _K^{l_1}f_1(z_K)\Vert _{S_{p_1}}^{p_1}d\nu (z) d\nu (y)dx\right) ^{1/p_1} \\&\quad =\left( \int _{{\mathbb {R}}}\int _{{\mathcal {V}}}\int _{{\mathcal {V}}}{\mathbb {E}}\Vert \sum _{K\in {\mathcal {D}}_{i,\kappa }}\varepsilon _K1_K(x)b_K(y_k,z_K)\Delta _K^{l_1}f_1(z_K)\Vert _{S_{p_1}}^{p_1}d\nu (z) d\nu (y)dx\right) ^{1/p_1} \\&\quad \le \left( \int _{{\mathbb {R}}}\int _{{\mathcal {V}}}\int _{{\mathcal {V}}}\max _{K\in {\mathcal {D}}_{i,\kappa }}|b_K(y_k,z_K)|{\mathbb {E}}\Vert \sum _{K\in {\mathcal {D}}_{i,\kappa }}\varepsilon _K1_K(x)\Delta _K^{l_1}f_1(z_K)\Vert _{S_{p_1}}^{p_1}d\nu (z) d\nu (y)dx\right) ^{1/p_1}. \end{aligned}$$

As in Case 1, we have the pointwise estimate $|b_K(y_k,z_K)|\le 1,$ since

$$\begin{aligned} |b_K(y_k,z_K)|&\le |K|^{3/2}\sum _{\begin{array}{c} L_1,L_3\in {\mathcal {D}}\\ L_j^{(l_j)}=K \end{array}}|b_{L_1,L_3,K}||h_{L_1}(z)||h_{L_3}(y_K)| \\&=|K|^{3/2}\sum _{\begin{array}{c} L_1,L_3\in {\mathcal {D}}\\ L_j^{(l_j)}=K \end{array}}|b_{L_1,L_3,K}|\frac{1_{L_1}(z)}{|L_1|^{1/2}}\frac{1_{L_3}(y_K)}{|L_3|^{1/2}} \\&\le |K|^{3/2}\sum _{\begin{array}{c} L_1,L_3\in {\mathcal {D}}\\ L_j^{(l_j)}=K \end{array}}\sum _{\begin{array}{c} Q_1,Q_2,Q_3\in {\mathcal {D}}\\ Q_j^{(k_j-l_j)}=L_j \end{array}}|a_{Q_1,Q_2,Q_3,K}|\prod _{j=1}^3\frac{|Q_j|^{1/2}}{|L_j|^{1/2}}\frac{1_{L_1}(z)}{|L_1|^{1/2}}\frac{1_{L_3}(y_K)}{|L_3|^{1/2}}\\&\le \sum _{\begin{array}{c} L_1,L_3\in {\mathcal {D}}\\ L_j^{(l_j)}=K \end{array}}\sum _{\begin{array}{c} Q_1,Q_2,Q_3\in {\mathcal {D}}\\ Q_j^{(k_j-l_j)}=L_j \end{array}}\prod _{j=1}^3\frac{|Q_j|}{|L_j|}{1_{L_1}(z)}{1_{L_3}(y_K)} \\&\le 1. \end{aligned}$$

Using the decoupling estimate (Theorem A.1) we thus conclude

$$\begin{aligned}&\left( \int _{{\mathbb {R}}}\int _{{\mathcal {V}}}\int _{{\mathcal {V}}}\max _{K\in {\mathcal {D}}_{i,\kappa }}|b_K(y_k,z_K)|{\mathbb {E}}\Vert \sum _{K\in {\mathcal {D}}_{i,\kappa }}\varepsilon _K1_K(x)\Delta _K^{l_1}f_1(z_K)\Vert _{S_{p_1}}^{p_1}d\nu (z) d\nu (y)dx\right) ^{1/p_1}\\&\quad \le \left( \int _{{\mathbb {R}}}\int _{{\mathcal {V}}}\int _{{\mathcal {V}}}{\mathbb {E}}\Vert \sum _{K\in {\mathcal {D}}_{i,\kappa }}\varepsilon _K1_K(x)\Delta _K^{l_1}f_1(z_K)\Vert _{S_{p_1}}^{p_1}d\nu (z) d\nu (y)dx\right) ^{1/p_1} \\&\quad =\left( {\mathbb {E}}\int _{{\mathbb {R}}}\int _{{\mathcal {V}}}\Vert \sum _{K\in {\mathcal {D}}_{i,\kappa }}\varepsilon _K1_K(x)\Delta _K^{l_1}f_1(z_K)\Vert _{S_{p_1}}^{p_1}d\nu (z)dx\right) ^{1/p_1} \le \beta _{{p_1},S_{p_1}}\int _{{\mathbb {R}}} \Vert f_1\Vert _{S_{p_1}}^{p_1}dx . \end{aligned}$$

The second case hence yields the estimate

$$\begin{aligned} \Vert S^k_i(f_1,f_2)\Vert _{L^p({\mathbb {R}},S_p)} \lesssim \beta _{p,S_p}^2 \beta _{p_1,S_{p_1}} \end{aligned}$$

in the case where the index of the non-cancellative Haar function is $j_0=2.$

Boundedness of the cases $j_0=1,3$ already follows from this result by cyclic permutation of the functions in the trilinear estimate (A.7) for $\Lambda _{S^k_i}.$ However, we can improve the resulting constant as follows.

**Case 2 is self-improving using cyclic permutations.** Let $j_0=1$ denote the index of the non-cancellative Haar function. By applying the decoupling estimate, Stein’s inequality, and Hölder’s inequality in the same manner as in the $j_0=2$ case, we obtain

$$\begin{aligned}  &   \Vert S_i^k(f_1,f_2)\Vert _{L^p({\mathbb {R}},S_p)} \le \beta ^2_{p,S_p} \Vert f_1\Vert _{L^{p_1}({\mathbb {R}},S_{p_1})} \\  &   \times ({\mathbb {E}}\int _{{\mathbb {R}}}\int _{{\mathcal {V}}}\Vert \sum _{K\in {\mathcal {D}}_{i,\kappa }}\varepsilon _K1_K(x)|K|^{1/2}\sum _{\begin{array}{c} L_2,L_3\in {\mathcal {D}}\\ L_j^{(l_j)}=K \end{array}}b_{L_2,L_3,K}\langle f_2,h_{L_2}\rangle h_{L_3}(y_K)\Vert _{S_{p_2}}^{p_2} d\nu (y)dx)^{1/p_2}. \end{aligned}$$

Proceeding to estimate the remaining integral as in the $j_0=2$ case yields

$$\begin{aligned} \Vert S_i^k(f_1,f_2)\Vert _{L^p({\mathbb {R}},S_p)} \le \beta ^2_{p,S_p} \beta _{p_2,S_{p_2}} \Vert f_1\Vert _{L^{p_1}({\mathbb {R}},S_{p_1})}\Vert f_2\Vert _{L^{p_2}({\mathbb {R}},S_{p_2})}. \end{aligned}$$

In order to optimise the behaviour of this constant as $p \searrow 1,$ we now apply the following permutation argument.

By writing out the trilinear form associated with $S_i^k,$ see (A.9), where we add the index of the non-cancellative Haar function as a superscript, we see that

$$\begin{aligned} \Lambda ^{(j_0=1)}_{S_i^k}(f_1,f_2,f_3)&= \sum _{K\in {\mathcal {D}}_{i,\kappa }}\sum _{\begin{array}{c} L_2,L_3\in {\mathcal {D}}\\ L_j^{(l_j)}=K \end{array}}b_{L_2,L_3,K}\tau \left( \langle f_1,h^0_{K}\rangle \langle f_2,h_{L_2}\rangle \langle f_3,h_{L_3}\rangle \right) \\  &= \sum _{K\in {\mathcal {D}}_{i,\kappa }}\sum _{\begin{array}{c} L_2,L_3\in {\mathcal {D}}\\ L_j^{(l_j)}=K \end{array}}b_{L_2,L_3,K}\tau \left( \langle f_3,h_{L_3}\rangle \langle f_1,h^0_{K}\rangle \langle f_2,h_{L_2}\rangle \right) \\&= \Lambda ^{(j_0=2)}_{S^{'k}_i}(f_3,f_1,f_2), \end{aligned}$$

where $S^{'k}_i$ is a dyadic shift with $j_0=2$ and the same scalar sequence $(b_{L_j,K})$ as $S_i^k$ up to renumbering. Noting that $\beta _{p^*,S_{p^*}}=\beta _{p,S_p}$ (see e.g. [23]), we can thus apply the estimate of the $j_0=2$ case to conclude

$$\begin{aligned} |\Lambda ^{(j_0=1)}_{S_i^k}(f_1,f_2,f_3)| = |\Lambda ^{(j_0=2)}_{S^{'k}_i}(f_3,f_1,f_2)| \lesssim \beta _{p_2,S_{p_2}}^2\beta _{p,S_p} \prod _{i=1}^3 \Vert f_i\Vert _{L^{p_i}({\mathbb {R}},S_{p_i})}, \end{aligned}$$

and by similar cyclic permutation arguments

$$\begin{aligned} |\Lambda ^{(j_0=2)}_{S_i^k}(f_1,f_2,f_3)|&= |\Lambda ^{(j_0=1)}_{S_i^{'k}}(f_2,f_3,f_1)| \lesssim \beta _{p_1,S_{p_1}}^2\beta _{p,S_p} \prod _{i=1}^3 \Vert f_i\Vert _{L^{p_i}({\mathbb {R}},S_{p_i})}, \\ |\Lambda ^{(j_0=3)}_{S_i^k}(f_1,f_2,f_3)|&= |\Lambda ^{(j_0=1)}_{S_i^{'k}}(f_3,f_1,f_2)| \lesssim \beta _{p_2,S_{p_2}}^2\beta _{p_1,S_{p_1}} \prod _{i=1}^3 \Vert f_i\Vert _{L^{p_i}({\mathbb {R}},S_{p_i})}, \\ |\Lambda ^{(j_0=3)}_{S_i^k}(f_1,f_2,f_3)|&= |\Lambda ^{(j_0=2)}_{S_i^{'k}}(f_2,f_3,f_1)| \lesssim \beta _{p_1,S_{p_1}}^2\beta _{p_2,S_{p_2}} \prod _{i=1}^3 \Vert f_i\Vert _{L^{p_i}({\mathbb {R}},S_{p_i})}, \end{aligned}$$

where $S_i^{'k}$ may denote different shifts in each line.

Combining all cases, where we consider all possible locations of the non-cancellative Haar function in Case 2, we conclude (using $\kappa _{2,q}\le 60,$ see Remark A.3)

$$\begin{aligned}  &   C({p,p_1,p_2})\lesssim \beta _{p}\beta _{p_1}\beta _{p_2} + +\min (\beta _{p_1}^2\beta _{p},\beta _{p}^2\beta _{p_1}) + \min (\beta _{p_2}^2\beta _{p},\beta _{p}^2\beta _{p_2})\\  &   +\min (\beta _{p_2}^2\beta _{p_1},\beta _{p_1}^2\beta _{p_2}), \end{aligned}$$

where we set $\beta _p:=\beta _{p,S_p}.$ Note that by [37], we have $\beta _{p,S_p}=pp^*,$ hence this notation agrees with the notation of the constant *C* in (6.9) used in the main body of this paper. $\square$
